# An Objective and Robust Bayes Factor for the Hypothesis Test One Sample and Two Population Means

**DOI:** 10.3390/e26010088

**Published:** 2024-01-20

**Authors:** Israel A. Almodóvar-Rivera, Luis R. Pericchi-Guerra

**Affiliations:** 1 Department of Mathematical Sciences, University of Puerto Rico at Mayagüez, Mayagüez, PR 00680, USA; 2 Department of Mathematics, University of Puerto Rico at Rio Piedras, San Juan, PR 00930, USA; luis.pericchi@upr.edu

**Keywords:** student’s *t* test, Bayes factors, intrinsic priors, robust prior

## Abstract

It has been over 100 years since the discovery of one of the most fundamental statistical tests: the Student’s *t* test. However, reliable conventional and objective Bayesian procedures are still essential for routine practice. In this work, we proposed an objective and robust Bayesian approach for hypothesis testing for one-sample and two-sample mean comparisons when the assumption of equal variances holds. The newly proposed Bayes factors are based on the intrinsic and Berger robust prior. Additionally, we introduced a corrected version of the Bayesian Information Criterion (BIC), denoted BIC-TESS, which is based on the effective sample size (TESS), for comparing two population means. We studied our developed Bayes factors in several simulation experiments for hypothesis testing. Our methodologies consistently provided strong evidence in favor of the null hypothesis in the case of equal means and variances. Finally, we applied the methodology to the original Gosset sleep data, concluding strong evidence favoring the hypothesis that the average sleep hours differed between the two treatments. These methodologies exhibit finite sample consistency and demonstrate consistent qualitative behavior, proving reasonably close to each other in practice, particularly for moderate to large sample sizes.

## 1. Introduction

One of the fundamental topics in statistics revolves around the one-sample population means and the comparison of two-sample means. The go-to method for addressing this question is typically the Student’s *t* test [1]. Conducting a hypothesis test for the population mean holds significant importance in the scientific research community and various fields where making inferences about population parameters is pivotal. Frequentists heavily rely on *p*-values to determine whether to reject or not reject the null hypothesis [2]. However, *p*-values, along with significance testing based on fixed α-levels, tend to exaggerate evidence against null hypotheses for large sample sizes and lack the operational meaning of a probability [3,4,5]. While the Bayesian approach has gained attention in hypothesis testing and model selection [6,7], its application in essential statistics topics remains somewhat limited [8]. This raises the question: Why is a Bayesian Student’s *t* test necessary? We argue for two main reasons. Firstly, Bayesian tests provide evidence for a hypothesis of interest that naturally adapts to any sample size. Secondly, the Bayes factor can be easily converted to posterior model probabilities and support one of the testing frameworks. Another crucial consideration is that scientific questions often have a Bayesian nature, such as, “What is the probability that these two treatments differ?”.

Bayesian hypothesis testing and model selection have been undergoing extensive development because of recent advances in the creation of ”default” Bayes factors that can be used in the absence of substantial subjective prior information [5,9,10,11]. The study in [12] proposed some arguments for the choice of the prior, such as (i) the fact that it is located around zero, (ii) the scale parameter σ, (iii) the fact that it is symmetric, and (iv) that it should have no moments. Bayes factors are attractive in terms of interpretation as odds, and the direct probability of the posterior model is readily understandable by general users of statistics [13]. Methods based on conjugate priors for the Student’s *t* test have a long history. Perhaps the most transparent approach for the two-sample Student’s *t* is in [14]. However, natural conjugate priors do not lead to robust procedures; they have tails that are typically of the same form as the likelihood function and will hence remain influential when the likelihood function is concentrated in the prior tails, which can lead to inconsistency [15]. This conjugate Bayes factor for comparing two samples based on the Student’s *t* is finite sample-inconsistent, i.e., it does not go to zero when the estimates go to infinity.

In this work, we proposed an objective and robust Bayes factor for testing the hypothesis of one-sample and two-sample means based on the *t*-statistic. Our Bayes factors can be easily implemented, allowing researchers to determine support for a particular hypothesis. This manuscript proceeds as follows. In Section 2, we derive these objectives and robust Bayes factors for one-sample and two-sample scenarios and demonstrate their finite sample consistency. In Section 3, we compare our Bayes factors with existing methodologies under several experimental frameworks. In Section 4, we apply our methodologies to real-life datasets such as the original Gosset sleep data and to comparisons of changes in blood pressure in mice according to their assigned diet. We conclude this work with a discussion in Section 5.

## 2. Methodology

Statistical inference for the mean (one or two samples) has an important rule in statistics and several fields. For instance, it is very common to test in terms of the average or population mean. Suppose that we are comparing two hypotheses, $H_{0}:\theta_{0}\in \Theta \;\mathrm{vs}.\;H_{1}:\theta_{1}\in \Theta$. Suppose that we have available prior densities $\pi_{i},i=1,2$ for each hypothesis and let  $f_{i}\left(x|\theta_{i}\right)$ be the probability density function under the *i*th hypothesis. Define the marginal or predictive densities for each hypothesis of interest (or model),

(1) $$m_{i}\left(x\right)=\int f_{i}\left(x|\theta_{i}\right)\pi_{i}\left(\theta_{i}\right)d\theta_{i},$$

which are sometimes called the evidence of the *i*th hypothesis or model. The Bayes factor for comparing $H_{0}$ to $H_{1}$ is then given by

(2) $$B_{01}=\frac{m_{0}\left(x\right)}{m_{1}\left(x\right)}=\frac{\int f_{0}\left(x|\theta_{0}\right)\pi_{0}\left(\theta_{0}\right)d\theta_{0}}{\int f_{1}\left(x|\theta_{1}\right)\pi_{1}\left(\theta_{1}\right)d\theta_{1}}.$$

The interpretation of the Bayes factor proceeds as follows. If $B_{01}>1$, then the evidence is in favor of the null hypothesis, while $B_{01}<1$ gives evidence in favor of the alternative hypothesis. If prior probabilities $P(H_{i})$i=0,1 of the hypotheses are available, then one can compute the posterior probabilities of it from the Bayes factors. The posterior probability of $H_{0}$, given the data x, is

(3) $$P\left(H_{0}|x\right)=\frac{m_{0}\left(x\right)P\left(H_{0}\right)}{\sum_{j=0}^{1}m_{j}\left(x\right)P\left(H_{j}\right)}=\frac{1}{1+\frac{P(H_{1})}{P(H_{0})}B_{10}};$$

where $B_{10}=1/B_{01}$.

### 2.1. One-Sample Mean Hypothesis Testing

A one-sample hypothesis test for the population mean is one of the most fundamental statistics topics, either as an introductory topic or to address research questions. Suppose we have a random sample from a normal distribution, i.e., $X_{1},\ldots ,X_{n}∼N\left(\mu ,\sigma^{2}\right)$, with an unknown standard deviation σ>0. We are interested in testing for the population mean μ.

(4) $$H_{0}:\mu =\mu_{0}\;\mathrm{vs}.\;H_{1}:\mu \neq \mu_{0}.$$

A Bayesian approach to test this hypothesis is based on the theory of intrinsic priors [16,17]. The authors begin with the noninformative priors for the null and alternative hypotheses, $\pi_{0}^{N}\left(\sigma \right)=1/\sigma$ and $\pi_{1}^{N}\left(\mu ,\sigma \right)=1/\sigma^{2}.$ After some calculations, the authors showed that the conditional proper intrinsic prior under the alternative Hypothesis $H_{1}$ is given by

$$\pi^{I}\left(\mu |\sigma \right)=\frac{1}{2\sqrt{\pi }\sigma }\frac{1-e^{-\mu^{2}/\sigma^{2}}}{\left(\mu^{2}/\sigma^{2}\right)}.$$

One can express $\pi^{I}\left(\mu ,\sigma \right)=\pi \left(\mu |\sigma \right)\pi \left(\sigma \right)$. The resulting intrinsic prior under $H_{1}$ is defined as

$$\pi_{1}^{I}\left(\mu ,\sigma \right)=\pi_{1}^{I}\left(\mu |\sigma \right)/\sigma =\frac{1}{2\sqrt{\pi }}\frac{1-e^{-\mu^{2}/\sigma^{2}}}{\mu^{2}}.$$

The approximate Bayes factors based on the intrinsic prior $\left(B_{01}^{IP}\right)$ for a one-sample population mean are

$$B_{01}^{IP}\approx B_{01}^{N}\cdot \frac{\pi_{1}^{N}\left(\hat{\mu },\hat{\sigma }\right)}{\pi_{1}^{I}\left(\hat{\mu },\hat{\sigma }\right)}\left(1+o\left(1\right)\right).$$

Here, $\hat{\mu }$ and $\hat{\sigma }$ are the Maximum Likelihood Estimators (MLEs) under $H_{1}$. The resulting Bayes factor for the hypothesis in (4) is

(5) $$B_{01}^{IP}\approx \sqrt{2n}{\left(1+\frac{t^{2}}{n-1}\right)}^{-n/2}\frac{t^{2}/\left(n-1\right)}{1-e^{-t^{2}/\left(n-1\right)}};$$

where $t=\left(\bar{x}-\mu_{0}\right)/s/\sqrt{n}$, where $\bar{x}$ is the sample mean and *s* is the sample standard deviation. Larges values of $B_{01}$ give evidence in favor of the null hypothesis. Also, we can transform these Bayes factors using the natural logarithm scale ($2\log B_{01}$), and values above 3 give some evidence in favor of the null hypothesis, while values above 10 give stronger evidence in favor of the null hypothesis; see [13].

This Bayes factor satisfies the finite sample consistency principle. Suppose that we are comparing the alternative hypothesis with the null hypothesis, $H_{0}:\beta =0$. As the least squares estimate $\hat{\beta }$ (and the noncentrality parameter) goes to infinity, so that one becomes sure that $H_{0}$ is wrong, the Bayes factor of $H_{0}$ to $H_{1}$ goes to zero.

**Theorem** **1.** *For a fixed sample size n≥2, the Bayes factor based on the intrinsic prior ($B_{01}^{IP}$) for the one-sample mean μ is finite sample-consistent.*

**Proof.**  For a fixed sample, n≥2, and letting $t^{2}\to \infty$ or equivalently |t|→∞, the Bayes factor based on the intrinsic prior goes to 0, i.e.,

$$\lim_{|t|\to \infty }B_{01}^{IP}\to 0;\mathrm{or}\;\mathrm{equivalently}\lim_{|t|\to \infty }P\left(H_{0}|x\right)\to 0.$$

The Bayes Factor based on the intrinsic prior $B_{01}^{IP}$ is finite sample consistent. □

#### Robust Bayes Factor for the One-Sample Test for the Mean

Even though the Bayes Factor constructed using the intrinsic prior is finite sample-consistent, it is only an approximation. Evidence has been found that priors with flatter tails than those of the likelihood function tend to be fairly robust, [18,19]. The robust prior proposed here is developed by [20]; we call it the Berger robust prior. This prior is hierarchical; by such a choice, we can obtain robustness while keeping the calculations relatively simple, and the computations are exact. The definition of this robust prior, denoted $\pi^{R}\left(\xi \right)$, can be defined as follows:

1. $\xi |\lambda ∼N_{p}\left(\mu ,B\left(\lambda \right)\right)$, where $B\left(\lambda \right)=\rho \lambda^{-1}\left(b+d\right)-d\;\mathrm{and}\;\rho =\frac{p+1}{p+3}.$
2. λ has a density $\pi \left(\lambda \right)=\frac{1}{2}\lambda^{-1/2}$ on (0,1).

where *p* is the rank of the design matrix. Recall that we are interested in testing (4); therefore, under the null hypothesis, the likelihood is in the form

$$f_{0}\left(x|\mu_{0},\sigma \right)={\left(2\pi \right)}^{-n/2}\sigma^{-n}\exp\left\{-\frac{\sum_{i=1}^{n}{\left(x_{i}-\mu_{0}\right)}^{2}}{2\sigma^{2}}\right\}.$$

The noninformative prior under the null hypothesis is $\pi^{N}\left(\sigma \right)=1/\sigma$. The marginal density m(x) under the null hypothesis is given by

(6) $$\begin{array}{cc} m_{0}\left(x\right) & =\int f_{0}\left(x|\sigma \right)\pi^{N}\left(\sigma \right)d\sigma =\int_{0}^{\infty }{\left(2\pi \right)}^{-n}\sigma^{-n}e^{-\frac{SS_{0}^{2}}{2\sigma^{2}}}\sigma^{-1}d\sigma \\ \; & ={\left(2\pi \right)}^{-n/2}{\left(SS_{0}^{2}\right)}^{-n/2}\Gamma \left(\frac{n}{2}\right); \end{array}$$

where $SS_{0}^{2}=\sum_{i=1}^{n}{\left(x_{i}-\mu_{0}\right)}^{2}$, the sums of squares under $H_{0}$ and Γ(·) is the gamma function. Similarly, we can obtain an alternative likelihood under the alternative Hypothesis $H_{1}$:

$$f_{1}\left(x|\mu ,\sigma \right)={\left(2\pi \right)}^{-n/2}\sigma^{-n}\exp\left\{-\frac{SS_{1}^{2}}{2\sigma^{2}}-\frac{n{\left(\mu -\bar{x}\right)}^{2}}{2\sigma^{2}}\right\}.$$

Here, $\bar{x}=n^{-1}\sum_{i=1}^{n}x_{i}$ is the sample mean and $SS_{1}^{2}=\sum_{i=1}^{n}{\left(x_{i}-\bar{x}\right)}^{2}$ is the sum of squares under the alternative. The Berger robust prior will be considered under the alternative hypothesis $\pi_{1}\left(\mu ,\sigma \right)=\pi^{R}\left(\mu |\sigma \right)/\sigma$. The marginal density under $H_{1}$ is

(7) $$\begin{array}{cc} m_{1}\left(x\right) & =\int f_{1}\left(x|\mu ,\sigma \right)\pi_{1}\left(\mu ,\sigma \right)d\mu d\sigma =\int f_{1}\left(x|\mu ,\sigma \right)\pi^{R}\left(\mu |\sigma \right)\frac{1}{\sigma }d\mu d\sigma \\ \; & =\frac{{\left(2\pi \right)}^{-n/2}}{n\cdot {\left(\bar{x}-\mu_{0}\right)}^{2}}\sqrt{\frac{n+1}{2}}\left(\frac{\Gamma ((n-2)/2)}{2{\left(\frac{s^{2}}{2}\right)}^{(n-2)/2}}-\frac{\Gamma ((n-2)/2)}{2{\left(\frac{s^{2}}{2}+\frac{{\left(\bar{x}-\mu_{0}\right)}^{2}}{(n+1)/n}\right)}^{(n-2)/2}}\right). \end{array}$$

Computing the ratio of the marginals from (6) and (7), the Bayes factor based on the Berger robust prior is given by

(8) $$B_{01}^{R}=\sqrt{\frac{2}{n+1}}\frac{n-2}{n-1}t^{2}{\left(1+\frac{t^{2}}{n-2}\right)}^{-n/2}{\left(1-{\left(1+\frac{2t^{2}}{n^{2}-1}\right)}^{-(n-2)/2}\right)}^{-1}.$$

Here, $t=\left(\bar{x}-\mu_{0}\right)/\left(s/\sqrt{n}\right)$ is the usual *t*-statistic with n−1 degrees of freedom, where $\bar{x}$ and *s* are the sample mean and sample standard deviation, respectively.

**Theorem** **2.** *For a fixed sample size n≥3, the Bayes factor based on the Berger robust prior ($B_{01}^{R}$) for the one-sample mean μ is finite sample-consistent.*

**Proof.**  For a fixed sample, n≥3, and letting $t^{2}\to \infty$, or equivalently |t|→∞, the Bayes factor based on the Berger robust prior goes to 0, i.e.,

$$\lim_{|t|\to \infty }B_{01}^{R}\to 0;\mathrm{or}\;\mathrm{equivalently}\lim_{|t|\to \infty }P\left(H_{0}|x\right)\to 0.$$

The Bayes factor based on the Berger robust prior $B_{01}^{R}$ is finite sample-consistent. □

Unlike the Bayes factor derived with the intrinsic prior, this robust Bayes factor has a closed form. We conclude the derivations for the Bayesian approach based on the intrinsic and robust prior that are finite sample-consistent. We now extend the objective and robust Bayesian approach to the two-sample scenarios.

### 2.2. Two-Sample Mean Hypothesis Test

Another fundamental research question of interest is whether or not the two groups are similar. This problem is usually addressed in the two-sample Student’s *t* test to compare if these groups differ in means. Let $X_{1},\ldots ,X_{n_{1}}∼N\left(\mu_{1},\sigma^{2}\right)$ and let $Y_{1},\ldots ,Y_{n_{2}}∼N\left(\mu_{2},\sigma^{2}\right)$ independent of *X* with σ>0 unknown. At first, we noticed that we were assuming that these two samples arise from a normal distribution with different means but equal variances. It is common interest to determine if these two samples are equal, or at least that they do not differ in location. To answer this, a hypothesis test for comparing two-sample means is performed, i.e., $H_{0}:\mu_{1}=\mu_{2}\;\mathrm{against}\;H_{1}:\mu_{1}\neq \mu_{2}$. To answer this question, Ref. [14] proposed the conjugate Bayes factor. This Bayes factor is based on the conjugate prior: $\left(\mu_{1}-\mu_{2}\right)/\sigma =\delta /\sigma |\sigma ∼N\left(\lambda ,\sigma_{\delta }^{2}\right).$ Centering the prior assessment on the null hypothesis, i.e., making λ=0, is usually a very reasonable choice. Then, the conjugate Bayes factor is simplified as $B_{01}^{C}={\left[\frac{1+t_{\nu }^{2}/\nu }{1+t_{\nu }^{2}/\left(\nu \left(1+n_{\delta }\sigma_{\delta }^{2}\right)\right)}\right]}^{-(\nu +1)/2}\sqrt{1+n_{\delta }\sigma_{\delta }^{2}}.$ However, this Bayes factor is not finite sample-consistent, as |t|→∞, or $t^{2}\to \infty$; the $B_{01}^{C}$ does not go to zero, or equivalently, the posterior probability of the null hypothesis $P(H_{0}|data)$ does not go to zero. In fact, as |t|→∞, then $B_{01}^{C}\to {\left(1+n_{\delta }\sigma_{\delta }^{2}\right)}^{-\nu /2}>0$, where $n_{\delta }=1/\left(1/n_{1}+1/n_{2}\right)$ and $\nu =n_{1}+n_{2}-2$ are the degrees of freedom.

#### 2.2.1. Intrinsic Bayes Factor for Two-Sample Means

To address the limitation of the conjugate prior, our first approach is based on the theory of intrinsic priors introduced in [16,17]. Similar to the one-sample case, the method is to dig out a prior that yields, for moderate to large sample sizes, results equivalent to an established method for scaling the intrinsic Bayes factors. The resulting set of equations typically has solutions, at least in the nested hypothesis scenario, which is our case, and has been successfully applied coupled with the intrinsic Bayes factor method. Consider the hypotheses tests for the comparison of two populations means with unknown and equal variance $\sigma^{2}>0$, $H_{0}:\mu_{1}=\mu_{2}\;\mathrm{vs}.\;H_{1}:\mu_{1}\neq \mu_{2}$. Let $\delta_{0}=\left(\mu_{1}+\mu_{2}\right)/2$ and $\delta_{1}=\left(\mu_{1}-\mu_{2}\right)/2$, then $\mu_{1}=\delta_{0}+\delta_{1}$ and $\mu_{2}=\delta_{0}-\delta_{1}$. This transformation leads us to the following design matrix X based on the training samples:

$$X\mu =\left[\begin{array}{cc} 1_{2\times 1} & 0_{2\times 1}\\ 0_{2\times 1} & 1_{2\times 1} \end{array}\right]\left[\begin{array}{c} \mu_{1}\\ \mu_{2} \end{array}\right]=\left[\begin{array}{cc} 1_{4\times 1}| & \left[\begin{array}{c} 1_{2\times 1}\\ -1_{2\times 1} \end{array}\right] \end{array}\right]\left[\begin{array}{c} \delta_{0}\\ \delta_{1} \end{array}\right]=\left[\begin{array}{cc} X_{0}\left(l\right): & X_{1}\left(l\right) \end{array}\right]\delta ;$$

where $1_{k\times 1}$ and $0_{k\times 1}$ are vectors of 1’s and 0’s of length *k*. The parameter of non-centrality can be computed as

(9) $$\begin{array}{cc} \lambda (l) & =\sigma^{-2}\delta_{1}^{t}X_{1}^{t}\left(l\right)\left(I-X_{0}\left(l\right){\left[X_{0}^{t}\left(l\right)X_{0}\left(l\right)\right]}^{-1}X_{0}^{t}\right)X_{1}\left(l\right)\delta_{1}\\ \; & =\sigma^{-2}{\left(\frac{\mu_{1}-\mu_{2}}{2}\right)}^{2}\cdot 4=\frac{{\left(\mu_{1}-\mu_{2}\right)}^{2}}{\sigma^{2}}; \end{array}$$

which becomes, in the comparison of two means, $\lambda \left(l\right)={\left(\mu_{1}-\mu_{2}\right)}^{2}/\sigma^{2}$. Following the general theory of the intrinsic Bayes factor for linear models [16,17], we have that an intrinsic prior is of the following form:

$$\pi^{I}\left(\lambda \left(l\right)|\sigma \right)=\frac{1}{\sqrt{2\pi }\sigma }\frac{\left(1-e^{-\lambda (l)/2}\right)}{\lambda (l)}.$$

Substitution from λ(l) using the non-centrality parameter of (9), then the transformation to the conditional of the parameter under the simple test, is $H_{0}:\delta_{1}=0\;\mathrm{vs}.\;H_{1}:\delta_{1}\neq 0$. The conditional intrinsic prior for the hypothesis test is

(10) $$\pi_{1}^{I}\left(\delta_{1}|\sigma \right)=\frac{1-e^{-8\delta_{1}^{2}/\sigma^{2}}}{4\sqrt{2\pi }\left(\delta_{1}^{2}/\sigma \right)}.$$

This conditional prior is proper, i.e., $\int \pi_{1}^{I}\left(\delta_{1}|\sigma \right)d\delta_{1}=1$ and it satisfies the condition discussed by [12]. The intrinsic prior, under the alternative Hypothesis $H_{1}$, is of the form $\pi_{1}^{I}\left(\delta_{1},\delta_{0},\sigma \right)=\pi_{1}^{I}\left(\delta_{1}|\delta_{0},\sigma \right)\cdot \pi_{1}^{I}\left(\delta_{0},\sigma \right)$, where $\pi_{1}^{I}\left(\delta_{0},\sigma \right)=1/\sigma$, i.e.,

(11) $$\pi_{1}^{I}\left(\delta_{0},\delta_{1},\sigma \right)=\frac{1}{\sigma }\cdot \frac{1-e^{-8\delta_{1}^{2}/\sigma^{2}}}{4\sqrt{2\pi }\delta_{1}^{2}/\sigma }=\frac{1-e^{-8\delta_{1}^{2}/\sigma^{2}}}{4\sqrt{2\pi }\delta_{1}^{2}}.$$

Setting up this framework, we can derive the intrinsic Bayes factor to compare two-sample means. We will first obtain the marginal density under the null hypothesis $m_{0}\left(x,y\right)$. First, consider the joint likelihood function of the two samples under the null Hypothesis $H_{0}$:

$$f_{0}\left(x,y|\delta_{0},\sigma \right)={\left(2\pi \right)}^{-n/2}\sigma^{-n}\exp\left\{-\frac{S_{x}^{2}+S_{y}^{2}+n_{1}{\left(\bar{x}-\delta_{0}\right)}^{2}+n_{2}{\left(\bar{y}-\delta_{0}\right)}^{2}}{2\sigma^{2}}\right\}.$$

Here, $\bar{x}$ is the sample mean of the first group, $\bar{y}$ is the sample mean of the first group, and $S_{x}^{2}=\sum_{i=1}^{n_{1}}{\left(x_{i}-\bar{x}\right)}^{2}$, $S_{y}^{2}=\sum_{i=1}^{n_{2}}{\left(y_{i}-\bar{y}\right)}^{2}$ are the sums of squares under the null hypothesis. The marginal density using the non-informative prior $\pi^{N}\left(\delta_{0},\sigma \right)=1/\sigma$ is computed as

(12) $$\begin{array}{cc} m_{0}\left(x,y\right) & =\int \int f_{0}\left(x,y|\delta_{0},\sigma \right)\pi \left(\delta_{0},\sigma \right)d\delta_{0}d\sigma \\ \; & =\frac{2^{(n-3)/2}\Gamma \left(\frac{n-1}{2}\right){\left(S_{x}^{2}+S_{y}^{2}\right)}^{-(n-1)/2}{\left(1+\frac{t^{2}}{n-2}\right)}^{-(n-1)/2}}{\sqrt{n}}, \end{array}$$

where $t=\left(\bar{x}-\bar{y}\right)/\left(S_{p}\sqrt{n_{\delta }}\right)$, where $n=n_{1}+n_{2}$, $S_{p}$ is the pooled standard deviation, i.e., $S_{p}^{2}=\left(S_{x}^{2}+S_{y}^{2}\right)/\left(n-2\right)$ and $n_{\delta }=1/n_{1}+1/n_{2}$. Similarly, the joint likelihood function of the two samples under the alternative Hypothesis $H_{1}$ is given by

$$f_{1}\left(x,y|\delta_{0},\delta_{1},\sigma \right)={\left(2\pi \right)}^{-n/2}\sigma^{-n}\exp\left\{-\frac{S_{x}^{2}+S_{y}^{2}+n_{1}{\left(\bar{x}-\left(\delta_{0}+\delta_{1}\right)\right)}^{2}+n_{2}{\left(\bar{y}-\left(\delta_{0}-\delta_{1}\right)\right)}^{2}}{2\sigma^{2}}\right\}.$$

The marginal density using the intrinsic prior $\pi^{I}\left(\delta_{0},\delta_{1},\sigma \right)$ defined in (11) is given by

(13) $$m_{1}\left(x,y\right)=\int \int \int f_{1}\left(x,y|\delta_{0},\delta_{1},\sigma \right)\pi^{I}\left(\delta_{0},\delta_{1},\sigma \right)d\delta_{0}d\delta_{1}d\sigma .$$

As in the one-sample framework, this Bayes factor can be approximated using the non-informative prior $\pi^{N}\left(\delta_{0},\delta_{1},\sigma \right)=1/\sigma^{2}$ in the asymptotic result as

(14) $$\begin{array}{cc} m_{1}\left(x,y\right) & \approx m_{1}^{N}\left(x,y\right)\frac{\pi^{I}\left({\hat{\delta }}_{0},{\hat{\delta }}_{1},\hat{\sigma }\right)}{\pi^{N}\left({\hat{\delta }}_{0},{\hat{\delta }}_{1},\hat{\sigma }\right)}\\ \; & =\int \int \int f_{1}\left(x,y|\delta_{0},\delta_{1},\sigma \right)\pi^{N}\left(\delta_{0},\delta_{1},\sigma \right)d\delta_{0}d\delta_{1}d\sigma \frac{\pi^{I}\left({\hat{\delta }}_{0},{\hat{\delta }}_{1},\hat{\sigma }\right)}{\pi^{N}\left({\hat{\delta }}_{0},{\hat{\delta }}_{1},\hat{\sigma }\right)}\\ \; & =\frac{\sqrt{\pi }2^{(n-3)/2-1/2}\Gamma \left(\frac{n-1}{2}\right){\left(S_{x}^{2}+S_{y}^{2}\right)}^{-(n-1)/2}}{\sqrt{n_{1}n_{2}}}. \end{array}$$

Using (12) and (14), we can compute $B_{01}^{N}$:

(15) $$B_{01}^{N}=\frac{m_{0}^{N}\left(x,y\right)}{m_{1}^{N}\left(x,y\right)}=\sqrt{\frac{2}{\pi n_{\delta }}}{\left(1+\frac{t^{2}}{n-2}\right)}^{-(n-1)/2};$$

where $t^{2}={\left(\bar{x}-\bar{y}\right)}^{2}/\left(S_{p}n_{\delta }\right)$, $S_{p}$ is the pooled estimate of the variance, $S_{p}^{2}=\left(S_{x}^{2}+S_{y}^{2}\right)/\left(n-2\right)$ and $n_{\delta }=\left(1/n_{1}+1/n_{2}\right)$. Let ${\hat{\delta }}_{1}$ and ${\hat{\sigma }}^{2}$ be the corresponding maximum likelihood estimator (MLE). Let ${\hat{\delta }}_{1}=\left(\bar{x}-\bar{y}\right)/2$ and ${\hat{\sigma }}^{2}=\left(S_{x}^{2}+S_{y}^{2}\right)/n=\left(n-2\right)/nS_{p}^{2}$; where $S_{p}^{2}$ is the variance pooled estimates and $n=n_{1}+n_{2}$. We can express the ${\hat{\delta }}_{1}^{2}/{\hat{\sigma }}^{2}=n_{\delta }nt^{2}/\left(n-2\right)$ in terms of the *t*-statistic. Then, the approximate intrinsic Bayes factor $B_{01}^{IP}$ can be obtained by

(16) $$\begin{array}{cc} B_{01}^{IP} & \approx B_{01}^{N}\frac{\pi^{N}\left({\hat{\delta }}_{0},{\hat{\delta }}_{1},\hat{\sigma }\right)}{\pi^{I}\left({\hat{\delta }}_{0},{\hat{\delta }}_{1},\hat{\sigma }\right)}=\sqrt{\frac{2}{\pi n_{\delta }}}{\left(1+\frac{t^{2}}{n-2}\right)}^{-(n-1)/2}\frac{{\hat{\delta }}_{1}^{2}/{\hat{\sigma }}^{2}}{\frac{1-e^{-8{\hat{\delta }}_{1}^{2}/{\hat{\sigma }}^{2}}}{4\sqrt{2\pi }}}\\ \; & =\frac{\sqrt{n_{\delta }}nt^{2}}{n-2}{\left(1+\frac{t^{2}}{n-2}\right)}^{-(n-1)/2}\left(1+\coth\left(\frac{n_{\delta }nt^{2}}{n-2}\right)\right). \end{array}$$

Here, $t=\left(\bar{x}-\bar{y}\right)/\left(Sp\sqrt{n_{\delta }}\right)$ and coth(·) is the hyperbolic cotangent function defined as $\coth\left(x\right)=\left(e^{2x}+1\right)/\left(e^{2x}-1\right)$.

**Theorem** **3.** *For a fixed sample size n≥4, the Bayes factor based on the intrinsic prior ($B_{01}^{IP}$) for the comparison of two population means is finite sample-consistent.*

**Proof.**  For a fixed sample, n≥4, and letting $t^{2}\to \infty$, or equivalently |t|→∞, the Bayes factor based on the intrinsic prior goes to 0, i.e.,

$$\lim_{|t|\to \infty }B_{01}^{IP}\to 0;\;\mathrm{or}\;\mathrm{equivalently}\lim_{|t|\to \infty }P\left(H_{0}|x\right)\to 0.$$

The Bayes factor based on the intrinsic prior $B_{01}^{IP}$ is finite sample-consistent. □

#### 2.2.2. Robust Bayes Factor for the Comparison of Two-Sample Means

Consider observations of a random sample from group 1 and group 2 of size $n_{1}$ and $n_{2}$, respectively. We assume these groups have common variance ($\sigma_{1}^{2}=\sigma_{2}^{2}=\sigma^{2})$, respectively. The model of interest in this case, $y_{ij}=\mu +\alpha_{i}+\varepsilon_{ij}$, with $\varepsilon_{ij}∼N\left(0,\sigma^{2}\right)$, for i=1,2 and $j=1,\ldots ,n_{i}$. We want to compare $H_{0}:\alpha_{1}=\alpha_{2}$ against $H_{0}:\alpha_{1}\neq \alpha_{2}$. Further, consider the constraint that $\alpha_{1}+\alpha_{2}=0$; then, the design matrix X can be written as

$$X=\left[\begin{array}{cc} 1_{n\times 1} & |\left[\begin{array}{c} 1/n_{1}1_{n_{1}\times 1}\\ -1/n_{2}1_{n_{2}\times 1} \end{array}\right] \end{array}\right];$$

This leads us to consider the following hypothesis, $H_{0}:\alpha_{1}=0\;\mathrm{vs}.\;H_{1}:\alpha_{1}\neq 0$. The reference’s priors, under the null hypothesis $H_{0}$ and alternative hypothesis $H_{1}$: $\pi_{0}^{N}\left(\mu ,\sigma \right)=1/\sigma$ and $\pi_{1}^{N}\left(\mu ,\alpha_{1},\sigma \right)=1/\sigma$. First, we proceed to find the marginal density under $H_{0}$. Consider the joint likelihood function under the null Hypothesis $H_{0}$:

$$f_{0}\left(y_{1},y_{2}|\mu ,\sigma \right)={\left(2\pi \right)}^{-n/2}\sigma^{-n}\exp\left\{-\frac{SS_{0}^{2}+n_{1}{\left({\bar{y}}_{1.}-\mu \right)}^{2}+n_{2}{\left({\bar{y}}_{2.}-\mu \right)}^{2}}{2\sigma^{2}}\right\}.$$

Here, $y_{1}=\left(y_{11},\ldots ,y_{1n_{1}}\right)$ and $y_{2}=\left(y_{21},\ldots ,y_{2n_{2}}\right)$, ${\bar{y}}_{i.}$ is the sample mean of the *i*th group, and $SS_{0}^{2}$ is the sum of squares under the null hypothesis. The marginal density under the null hypothesis $m_{0}\left(y_{1},y_{2}\right)$ is given by

(17) $$\begin{array}{cc} m_{0}\left(y_{1},y_{2}\right) & =\int \int f_{0}\left(y|\mu ,\sigma \right)\sigma^{-1}\;d\mu d\sigma \\ \; & =\frac{\Gamma ((n-1)/2)}{\sqrt{n}}\frac{{\left(\pi \right)}^{-(n-1)/2}}{2}{\left(SS_{0}^{2}\right)}^{-(n-1)/2}{\left(1+\frac{t^{2}}{n-2}\right)}^{-(n-1)/2}. \end{array}$$

where $t^{2}={\left({\bar{y}}_{1.}-{\bar{y}}_{2.}\right)}^{2}/\left(S_{p}n_{\delta }\right)$. Here, $S_{p}$ is the sample pooled estimate of the variance, $S_{p}^{2}=\left(S_{1}^{2}+S_{2}^{2}\right)/\left(n-2\right)$, and $n_{\delta }=\left(1/n_{1}+1/n_{2}\right)$. For the alternative Hypothesis $H_{1}$, we first consider the joint likelihood of group 1 and group 2:

$$f_{1}\left(y_{1},y_{2}|\mu ,\alpha ,\sigma \right)={\left(2\pi \right)}^{-n/2}\sigma^{-n}\exp\left\{\frac{\left(S_{1}^{2}+S_{2}^{2}+n_{1}{\left({\bar{y}}_{1.}-\left(\mu +\alpha \right)\right)}^{2}+n_{2}{\left({\bar{y}}_{2.}-\left(\mu -\alpha \right)\right)}^{2}\right)}{2\sigma^{2}}\right\}.$$

The marginal density $m_{1}\left(y_{1},y_{2}\right)$ is given by

(18) $$\begin{array}{cc} m_{1}\left(y_{1},y_{2}\right) & =\int \int \int f_{1}\left(y|\mu ,\alpha ,\sigma \right)\times \sigma^{-1}\pi^{R}\left(\alpha \right)\;d\mu d\alpha d\sigma \\ \; & =\sqrt{\frac{n_{\delta }\left(b+d\right)}{{\left(2\pi \right)}^{\left(n-2\right)}}}\frac{\Gamma ((n-3)/2)}{4\sqrt{\pi }n{\hat{\alpha }}^{2}}\left[{\left(\frac{S_{1}^{2}+S_{2}^{2}}{2}\right)}^{-(n-3)/2}-{\left(\frac{S_{1}^{2}+S_{2}^{2}}{2}+\frac{{\hat{\alpha }}^{2}}{b+d}\right)}^{-(n-3)/2}\right]. \end{array}$$

Here, $\hat{\alpha }=\left({\bar{y}}_{1.}-{\bar{y}}_{2.}\right)/2$. The robust Bayes factor is obtained by computing the ratio of the marginal densities of (17) and (18):

(19) $$B_{01}^{R}=\sqrt{\frac{8n_{\delta }}{b+d}}\left(\frac{t^{2}\left(n-3\right)}{4(n-2)}\right){\left(1+\frac{t^{2}}{n-2}\right)}^{-(n-1)/2}{\left(1-{\left(1+\frac{t^{2}n_{\delta }}{2(n-2)(b+d)}\right)}^{-(n-3)/2}\right)}^{-1}.$$

To finish the calculation of the robust Bayes factor $B_{01}^{R}$, the term b+d has to be defined. Therefore, we propose using the effective sample size (TESS) $n_{o}^{e}$ of [21]. The first factor $d=0.25n_{\delta }\cdot \sigma^{2}$, and the second factor *b* is $b=n_{o}^{e}\cdot d=0.25\sigma^{2}{\left(\max\left\{\left|\frac{1}{n_{1}}\right|,\left|-\frac{1}{n_{2}}\right|\right\}\right)}^{-2}$; then, $b+d=0.25\sigma^{2}\times \left[n_{\delta }+{\left(\max\left\{\left|\frac{1}{n_{1}}\right|,\left|-\frac{1}{n_{2}}\right|\right\}\right)}^{-2}\right]=0.25\sigma^{2}\times \left(d^{*}+b^{*}\right)$. Derivation of TESS is displayed in Appendix A.1.

**Theorem** **4.** *For a fixed sample size n ≥ 4, the Bayes factor based on the robust prior ($B_{01}^{R}$) for the comparison of two populations means is finite sample-consistent.*

**Proof.**  For a fixed sample, n≥4, and letting $t^{2}\to \infty$, or equivalently |t|→∞, the Bayes factor based on the Berger robust prior goes to 0, i.e.,

$$\lim_{|t|\to \infty }B_{01}^{R}\to 0;\;\mathrm{or}\;\mathrm{equivalently}\lim_{|t|\to \infty }P\left(H_{0}|x\right)\to 0.$$

The Bayes factor based on the Berger robust prior $B_{01}^{R}$ is finite sample-consistent. □

The Berger robust prior yields the following (exact) expression for the correction of the main term (for group *i*):

$$\pi^{R}\left(\xi_{i}|d_{i},b_{i}\right)=\frac{1}{\sqrt{2\pi (d_{i}+b_{i})}}\frac{\left[1-\exp\left(-\frac{\xi_{i}^{2}}{\left(d_{i}+b_{i}\right)}\right)\right]}{\sqrt{2}\xi_{i}^{2}/\left(d_{i}+b_{i}\right)}.$$

Making the change of variables $\xi_{i}=\sqrt{8(d_{i}+b_{i})}\;\;\beta^{*}/\sigma$ and η=σ then taking the Jacobian,

$$\left|J\right|=\left|\begin{array}{cc} \frac{\sqrt{8(d_{i}+b_{i})}}{\sigma } & \frac{-\sqrt{8(d_{i}+b_{i})}\beta^{*}}{\sigma^{2}}\\ 0 & 1 \end{array}\right|=\left|\frac{\sqrt{8(d_{i}+b_{i})}}{\sigma }\right|.$$

the conditional intrinsic prior of Equation (10) is exactly recovered; $\pi^{I}\left(\beta^{*}|\sigma \right)=\pi^{R}\left(\xi_{i}|d_{i},b_{i}\right)\left|J\right|$. This established a correspondence between the intrinsic and Berger’s robust priors for the Student’s *t* test.

#### 2.2.3. The Effective Sample Size Bayesian Information Criterion (BIC-TESS)

Our final Bayes factor for comparing two-sample means is a variation of the Bayesian Information Criterion (BIC) of [22]. The BIC is a popular method to determine the best model in a set of competing models. However, in comparing the two-sample means, the BIC does not consider the information available in both groups but rather the entire sample. Here, we proposed replacing the sample size *n* with TESS. This may be used to form what may be claimed to be the corrected BIC or BIC-TESS. It can be demonstrated that BIC with TESS is:

(20) $$B_{01}^{TESS}=\sqrt{n_{o}^{e}}{\left(1+\frac{t^{2}}{n-2}\right)}^{-n/2},$$

where $n_{o}^{e}$ is defined by [11]. Derivation of TESS is in Appendix A.1. If we have a balanced situation, where $n_{1}=n_{2}$, the BIC-TESS is similar to the regular BIC. If the situation is unbalanced, the BIC-TESS is stabilized, since as $n_{2}\to \infty$, the $B_{01}^{TESS}\to \sqrt{n_{1}}$.

**Theorem** **5.** *For a fixed sample size n≥3, the corrected BIC ($B_{01}^{TESS}$) for the two-sample mean μ is finite sample-consistent.*

**Proof.**  For a fixed sample, n≥3, and letting $t^{2}\to \infty$, or equivalently |t|→∞, the Bayesian Information Criterion constructed with TESS goes to 0, i.e.,

$$\lim_{|t|\to \infty }B_{01}^{TESS}\to 0;\;\mathrm{or}\;\mathrm{equivalently}\lim_{|t|\to \infty }P\left(H_{0}|x\right)\to 0.$$

The corrected Bayesian Information Criterion is $B_{01}^{TESS}$ is finite sample-consistent. □

**Figure 1:**
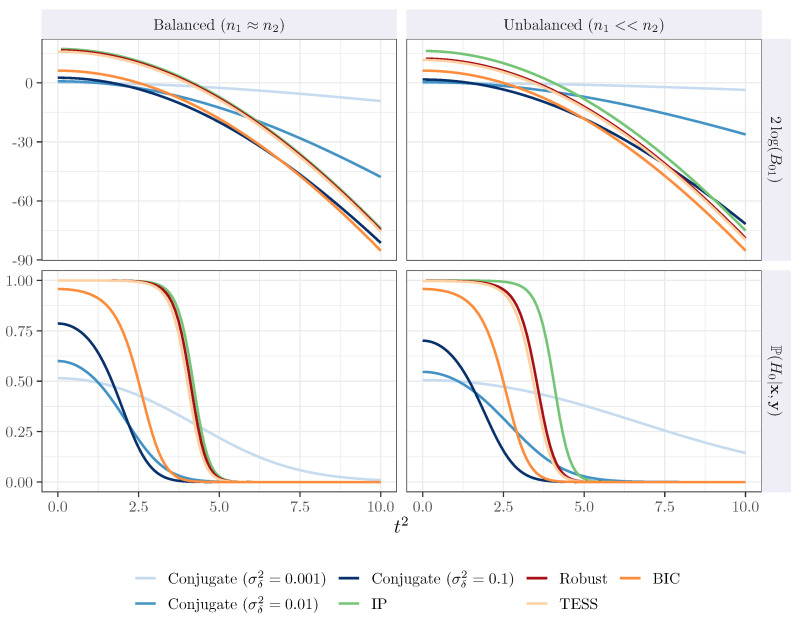
Results in terms of $2\log(B_{01})$ and posterior probability for the finite sample consistency.

In Figure 1, we compare the asymptotic behavior of the Bayes factors and the posterior probability of the null hypothesis when the samples are balanced ($n_{1}\approx n_{2}$) and unbalanced ($n_{1}<<n_{2})$. The Bayes factor, based on the Berger robust prior (dark red), is very close in the range of evidence to the intrinsic Bayes factor (green) and the BIC-TESS (light orange). The robust Bayes factor, the intrinsic Bayes factor, and the BIC-TESS are relatively closed when the situation is balanced. In the unbalanced scenario, the robust Bayes factor and the BIC-TESS remain relatively close, while the intrinsic Bayes factor slightly increases. The conjugate Bayes factor (blue) is represented with different values of the prior variance $\sigma_{\delta }^{2}$; darker color means higher values for the prior variance. Recall that the conjugate Bayes factor is not finite sample-consistent, and its behavior depends on the choice of $\sigma_{\delta }^{2}$.

## 3. Simulation Experiments

### Experiments for the One- and Two-Sample Mean Comparisons

We generated 500 datasets from random samples taken from a normal distribution, Student’s *t* distribution with one degree of freedom, and gamma distribution. For each of these distributions, the mean and standard deviation values were set to $\mu_{1}=5$ and $\sigma_{1}=3$. The second group was created with a combination of several parameters for the location; the mean values were $\mu_{2}\in \left\{\mu_{1},1.5\mu_{1},2\mu_{1}\right\}$, and for the standard deviation of the second group, $\sigma_{2}\in \left\{\sigma_{1},2\sigma_{1},3\sigma_{1}\right\}$. In the case of the Student’s *t* distribution, both groups were simulated with ν=1 degrees of freedom. The simulated gamma samples were obtained using the method of moments for the shape parameter, with $\alpha_{i}=\mu_{i}^{2}/\sigma_{i}^{2}$, and the scale parameter, with $\beta_{i}=\sigma_{i}^{2}/\mu_{i}$, for i=1,2.

**Figure A1:**
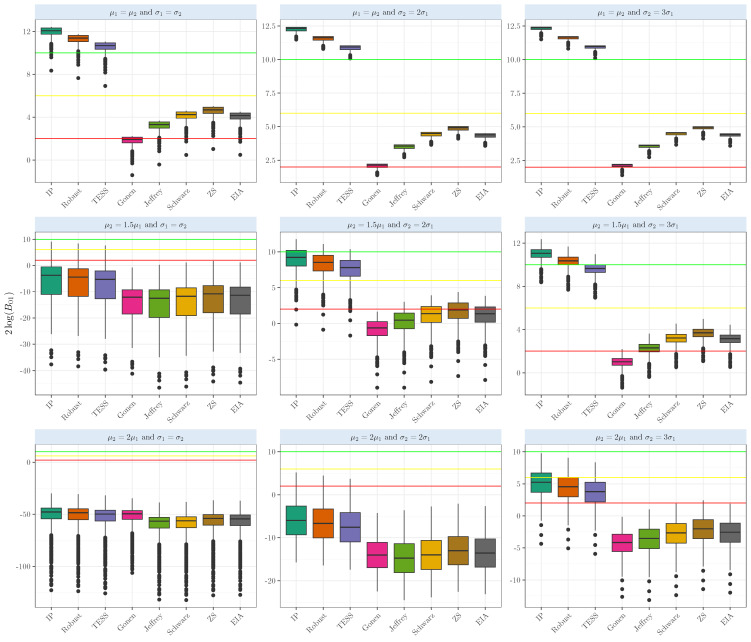
Evidence in the $2\log(B_{01})$ scale when comparing the population means of two samples that arise from normal distributions with several means and variances with equal sizes $n_{1}=50$ and $n_{2}=500$. Intrinsic priors (IP), Berger robust prior (Robust), BIC based on the effective sample size (TESS), conjugate, Jeffrey’s, Schwarz, Zellner and Siow (ZS), and the expected arithmetic intrinsic prior of [24].

**Figure A2:**
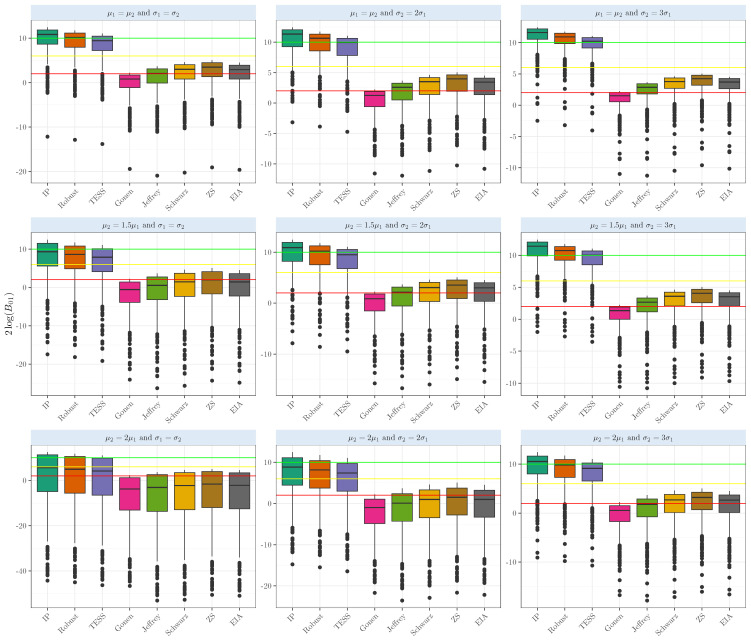
Evidence in the $2\log(B_{01})$ scale when comparing the population means of two samples that arise from a Student’s *t* distributions with several means and variances with equal sizes $n_{1}=50$ and $n_{2}=500$. Intrinsic priors (IP), Berger robust prior (Robust), BIC based on the effective sample size (TESS), conjugate, Jeffrey’s, Schwarz, Zellner and Siow (ZS), and the expected arithmetic intrinsic prior of [24].

**Figure A3:**
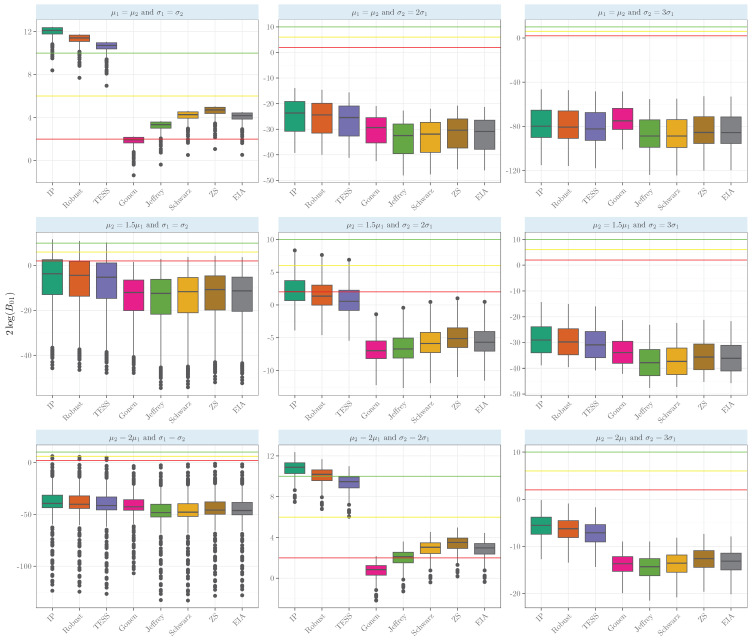
Evidence in the $2\log(B_{01})$ scale when comparing the population means of two samples that arise from gamma random samples with several means and variances with equal sizes $n_{1}=50$ and $n_{2}=500$. Intrinsic priors (IP), Berger robust prior (Robust), BIC based on the effective sample size (TESS), conjugate, Jeffrey’s, Schwarz, Zellner and Siow (ZS), and the expected arithmetic intrinsic prior of [24].

We compared our methodologies with several Bayes factors used when comparing two population means, displayed in Table 1. $B_{01}^{S}$ is the classical Bayesian Information Criterion (BIC) of [22], ($B_{01}^{ZS}$) is based on the Zellner and Siow prior [23], the two-sample Student’s *t* Bayes factor of [14] is based on the conjugate prior with $\sigma_{\delta }^{2}=1/3$, the arithmetic Bayes factor ($B_{10}^{EIA}$) of [24], and [12]’s Bayes Factor ($B_{01}^{J}$) for the comparison of two-sample means with equal variances. One set of these Bayes factors—the BIC of Schwartz and the Zellner and Siow Bayes factors—depends only on the sample size *n*. The other set, based on the conjugate prior, intrinsic, Berger’s (here called robust), and finally, the modified Jeffrey’s prior, depends on the term $n_{\delta }=1/n_{1}+1/n_{2}$. In our experiments, we do not consider the constant 2/5 for $B_{01}^{J}$, since we believe it satisfies the condition that the samples arise from the same distribution; for more details about the use of the constant 2/5, see [12]. We also studied these Bayes factors in unbalanced situations. Heavily unbalanced samples are interesting not only from a theoretical point of view but also because they are often observed in practice in observational studies; the results of these are displayed in Figure A1, Figure A2 and Figure A3.

Performance was compared using the twice natural base logarithm Bayes factors $\left(2\log\left(B_{01}\right)\right)$ for comparing the null hypothesis ($\mu_{1}=\mu_{2}$) against the alternative ($\mu_{1}\neq \mu_{2}$). This transformation allows the interpretation to be on the same scale as the deviance and likelihood ratio test statistics, as discussed in [13].

**Figure 2:**
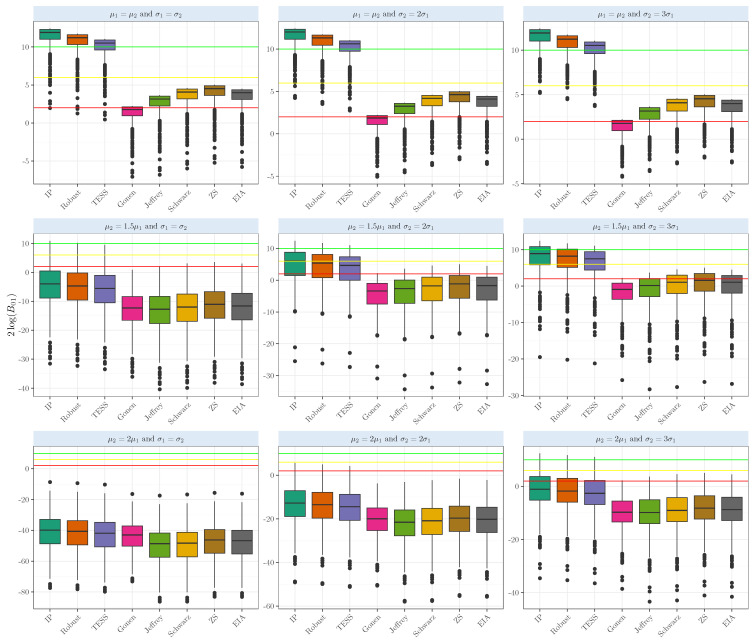
Evidence in the $2\log(B_{01})$ scale when comparing the population means of two samples that arise from a normal distribution with several means and variances with equal sizes $n_{1}=n_{2}=50$.

Figure 2 displays the results for the evidence based on the normal distributions when testing whether two-sample means are equal ($\mu_{1}=\mu_{2}$). The red line represents the cut-off for 10 (strong evidence), the yellow line for 6 (positive evidence), and the green for 2 (weak evidence). In the actual case when the means are equal, the Bayes factors based on the intrinsic prior and robust prior show strong evidence in favor of the null hypothesis. The average $2\log(B_{01})$ based on the intrinsic prior shows strong evidence in favor of the null hypothesis (11.3±1.59), while the Bayes factor based on the robust prior gives strong evidence in favor of the true case (10.61±1.59), all above the red line. BIC-TESS also strongly supports the true case (9.9±1.61). The other Bayes factors provide positive evidence for the true case, with averages ranging from 2.54 to 3.93. Even when the means were equal, and the samples had larger variance ($\sigma_{2}=3\sigma_{1}$), our objectives and robust Bayes factors provided strong evidence in favor of the true case, with the average above 10. The intrinsic Bayes factor and the robust prior were above 90%, showing either strong or very strong evidence in favor of the null hypothesis when the means were equal; see Table A2 for a detailed comparison.

**Figure 3:**
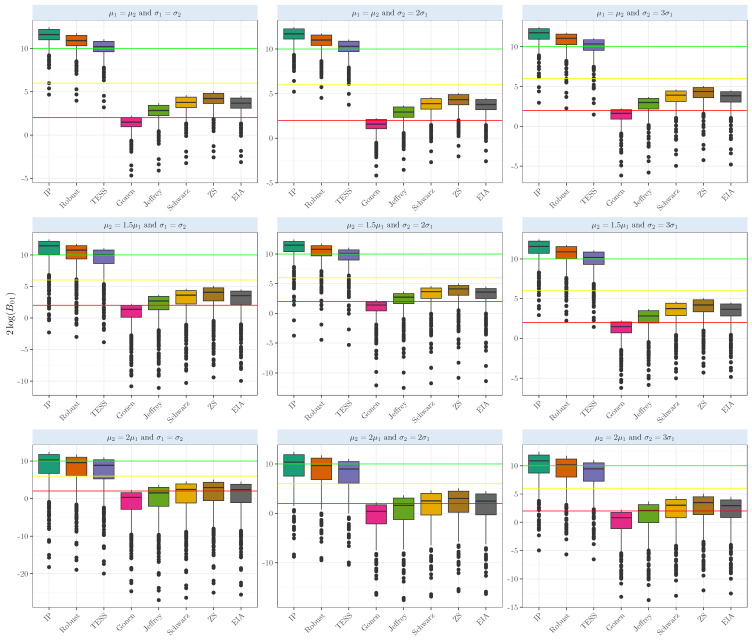
Evidence in the $2\log(B_{01})$ scale when comparing the population means of two samples that arise from a Student’s *t* with one degree of freedom with several means and variances with equal sizes $n_{1}=n_{2}=50$.

**Figure 4:**
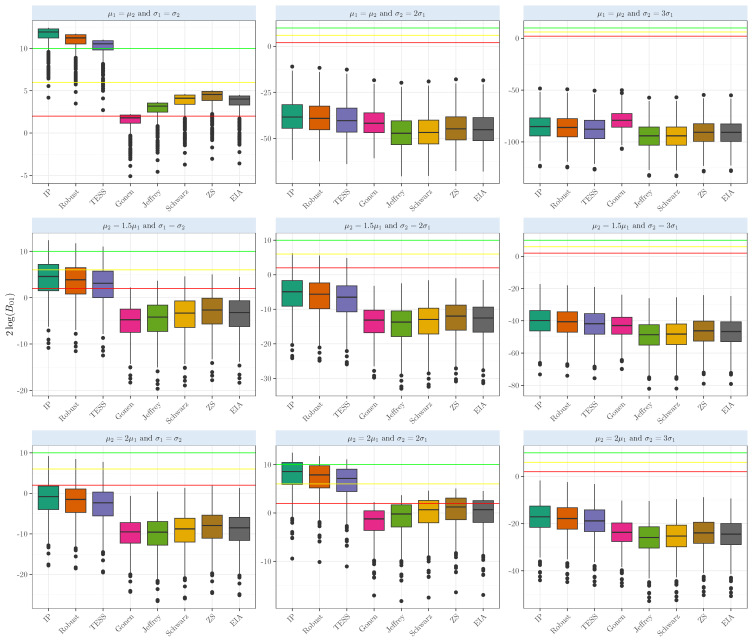
Evidence in the $2\log(B_{01})$ scale when comparing the population means of two samples that arise from a gamma distribution with several shapes and scales with equal sizes $n_{1}=n_{2}=50$.

In the Student’s *t* random samples, when testing whether two-sample means are equal ($\mu_{1}=\mu_{2}$), we can observe in Figure 3 the results when the means are equal. The Bayes Factors based on the intrinsic prior and robust prior show strong evidence in favor of the null hypothesis. The average $2\log(B_{01})$ based on the intrinsic prior, robust prior, and BIC-TESS shows strong evidence in favor of the null hypothesis (averages above 10), with values of 11.41,10.72, and 10.02; dispersion was relatively low, ranging from 1.01 to 1.03. The competing Bayes factors provide slightly positive evidence for the true case, with averages ranging from 2.65 to 4.04. It is interesting to see that in the case of $\mu_{2}=2\mu_{1}$, our Bayes factor gave positive evidence above the yellow line but was very variable; the sample standard deviation ranged from 4.98 to 5.03. Finally, in the case of gamma samples, our Bayes factors gave strong evidence only when the means and the variances were equal. Departing from any of these conditions gave strong evidence that the means were unequal; see Figure 4. For more details about the simulation results’ numerical performance, see Table A1.

## 4. Application in Real Dataset

In this section, we applied the proposed one and two Bayes factors based on the intrinsic, Berger, and robust priors, and BIC-TESS based on the Student’s *t* statistic.

### 4.1. Gosset Original Dataset

We first consider the century-long original Student’s *t* sleep data from [1,25] that still raise interesting discussion; see [26,27]. In this study, the number of hours of sleep under both drugs (Dextro and Laevo) was recorded for each patient. The difference in hours was recorded to determine effectiveness, and the average number of hours of sleep gained by using each drug (Dextro and Laevo) was measured. The authors concluded that, in usual doses, Laevo was soporific, but Dextro was not. This analysis is treated as a paired sample, since it compares the sleep hours between treatments. Paired samples lead us to the one sample. The hypothesis of interest is $H_{0}:\mu_{d}=0$ versus $H_{1}:\mu_{d}\neq 0$; the test statistic is t=−4.06 with a *p*-value of 0.002. At the 5% significance level, we can conclude that there is a difference in the average sleep hours between Laevo and Dextro.

However, the Gosset original dataset has not been addressed using an objective and robust Bayesian approach. The value of the test statistics is the same as before, with n=10. The ($2\log(B_{10})$) was computed for the intrinsic and robust Bayes factor, along with the associated posterior probabilities ($P(H_{1}|data)$). The $2\log(B_{10}^{IP})=5.858$ and $2\log(B_{10}^{R})=5.988$ are positive, indicating strong evidence that the average sleep hours are different. Further, the posterior probability based on the intrinsic prior is 0.949, and the posterior probability based on the Berger robust prior is 0.952. Both posterior probabilities are above 90%, suggesting strong evidence favoring the average sleep difference.

This dataset is considered as a paired sample, since the recorded number of sleep hours belongs to the same participant. However, the treatments, Dextro and Laevo, might need to be considered independently. If these are considered independently, then a two-sample framework arises. We are interested in determining the sleep hours when receiving Laevo versus when receiving Dextro. Assuming equal variances between Laevo and Dextro, the hypothesis of interest is $H_{0}:\mu_{L}=\mu_{D}$ versus $H_{1}:\mu_{L}\neq \mu_{D}$, where $\mu_{L}$ is the average sleep hours when receiving Laevo and $\mu_{D}$ is the average sleep hours when receiving Dextro. The two-sample test statistic is t=−1.86 with a *p*-value of 0.079. At the 5% significance level, we can conclude that there is no difference in the average sleep hours when using Laevo versus Dextro. In our Bayesian approach, $2\log(B_{10}^{IP})=-4.33$ and $2\log(B_{10}^{R})=-3.57$, indicating weak evidence that the average number of sleep hours differs between Laevo and Dextro. Both posterior probabilities are above 15%, suggesting weak evidence that the average number of sleep hours differs when using Laevo and Dextro.

### 4.2. Induced Hypertension on Mice According to Diet

Our first application consists of the data from [28], but they were analyzed in a Bayesian framework using intrinsic priors by [24]. In this study, the researchers were interested in how intermittent feeding affected the blood pressure of rats. The treatment group consisted of eight rats fed intermittently for weeks, and at the final period, the rats’ blood pressure measurements were taken. The blood pressure measurements of a second group of seven rats fed the usual way were defined as a control group. The hypothesis of interest is that the average blood pressure is different when the rats have intermittent fasting compared to those with their usual diet, i.e., $H_{0}:\mu_{1}=\mu_{2}$ versus $H_{1}:\mu_{1}\neq \mu_{2}$. At the 5% significance level, with a *p*-value =0.044, one can conclude that there exists a difference in the mean blood pressure level according to their feeding style.

The study in Ref. [24] computed the expected arithmetic Bayes factor that favors the alternative hypothesis $B_{10}^{EAI}=2.035$ with $P(H_{1}|\left(x,y\right))=0.671$, providing support that the average blood pressure measurements differ based on diet. Notably, the Bayes factors based on the intrinsic priors and robust priors yield negative values, $2\log(B_{10}^{IP})=-2.412$ and $2\log(B_{10}^{R})=-1.414$, respectively, indicating evidence against $H_{1}$. However, the corresponding posterior probabilities ($P(H_{1}|x,y)$) are 0.23 and 0.33, suggesting weak evidence for the alternative hypothesis that the means are different. The corrected BICTESS suggests weaker evidence against $H_{1}$ with a $2\log(B_{10}^{TESS})=-0.3634$ and a posterior probability of $P(H_{1}|x,y)=0.455$. In contrast, the conjugate $2\log(B_{10}^{C})=1.517$ and $2\log(B_{10}^{EAI})=1.421$, indicating very weak evidence in favor of $H_{1}$. The associated posterior probability is 0.681.

The extreme observation in the intermittent group (115) was removed. The $2\log(B_{10}^{IP*})=2.347$, suggesting evidence in favor of $H_{1}$, while the posterior probability of $P(H_{1}|\left(x^{*},y^{*}\right))=0.764$ indicates a moderate level of confidence in this conclusion. The Bayes factor constructed with the Berger robust prior exhibits a higher $2\log(B_{10}^{R*})=3.535$, along with the posterior probability of $P(H_{1}|\left(x^{*},y^{*}\right))=0.854$, indicating stronger support that the average of blood pressure differs by type of fasting. TESS models present even higher $2\log(B_{10}^{TESS*})=5.041$, respectively, with corresponding posterior probabilities of 0.926, indicating substantial evidence for $H_{1}$.

## 5. Discussion

In this work, we proposed the objective and robust Bayes factors for the one-sample and two-sample comparisons. These newly proposed Bayes factors are finite sample-consistent. Both the exact and approximate forms of the Bayes factors can be easily implemented using any open-source or commercial software. Another advantage of using Bayes factors is that the posterior probabilities of the hypothesis test are easily interpretable. We reanalyzed the original study by [1] and the comparison of blood pressure in rats according to different feeding types. Our objective and robust Bayes factors showed strong evidence that the average number of hours differed between Laevo and Dextro in the mouse application. When removing potential extreme values, we concluded that there is strong evidence that the means differed. However, we reported weak evidence with the complete dataset that these averages differed according to their diet. This might occur, since the assumption of equal variances might not hold. Even though the samples might have equal means, departing from the assumption of equal variances can lead in favor of the wrong hypothesis. Although we have made a significant contribution, an aspect that might alleviate this issue is deriving an objective and robust Bayes factor for the Behrens–Fisher problem, i.e., unequal variances for both groups. Also, the Bayes factor based on the intrinsic prior depends on the maximum likelihood estimate (MLE); perhaps robust estimates can be considered, although a modified test statistic might arise. Another possible extension is to develop an objective Bayes factor for the hypothesis of several equal means; this will be an analysis of variance (ANOVA) approach in the frequentist approach.

## Figures and Tables

**Table 1:** **Bayes factors based on the one- and two-sample means based on the Student’s *t* test. The third column applies only to the two-sample comparison and is limiting when $t^{2}\to 0$ and $n_{2}\to \infty$**.

One Sample	$B_{01}^{IP}\approx$	$\sqrt{2n}{\left(1+\frac{t^{2}}{n-1}\right)}^{-\frac{n}{2}}\frac{t^{2}}{n-1}{\left(1-e^{-t^{2}/\left(n-1\right)}\right)}^{-1}$	-
	$B_{01}^{R}=$	$\sqrt{\frac{2}{n+1}}\frac{n-2}{n-1}t^{2}{\left(1+\frac{t^{2}}{n-1}\right)}^{-\frac{n}{2}}{\left(1-{\left(1+\frac{2t^{2}}{n^{2}-1}\right)}^{-\frac{n-2}{2}}\right)}^{-1}$	-
Two Samples	$B_{01}^{IP}\approx$	$\frac{\sqrt{n_{\delta }}nt^{2}}{n-2}{\left(1+\frac{t^{2}}{n-2}\right)}^{-(n-1)/2}\left(1+\coth\left(\frac{n_{\delta }nt^{2}}{n-2}\right)\right)$	$\sqrt{n_{1}}$
	$B_{01}^{R}=$	$\sqrt{\frac{8n_{\delta }}{b+d}}\left(\frac{t^{2}\left(n-3\right)}{4(n-2)}\right){\left(1+\frac{t^{2}}{n-2}\right)}^{-(n-1)/2}{\left(1-{\left(1+\frac{t^{2}n_{\delta }}{2(n-2)(b+d)}\right)}^{-(n-3)/2}\right)}^{-1}$	$\sqrt{2(1+n_{1}^{3})}$
	$B_{01}^{TESS}=$	$\sqrt{n_{o}^{e}}{\left(1+\frac{t^{2}}{n-2}\right)}^{-n/2}$	$\sqrt{n_{1}}$
	$B_{01}^{J}\approx$	$\frac{2}{5}\sqrt{\frac{\pi n_{\delta }}{2}}{\left(1+\frac{t^{2}}{n-2}\right)}^{-(n-1)/2}$	$\frac{2}{5}\sqrt{\frac{\pi n_{1}}{2}}$
	$B_{01}^{C*}=$	${\left[\frac{1+t^{2}/\left(n-2\right)}{1+t^{2}/\left(\left(n-2\right)\right)\left(1+\sigma_{\delta }^{2}n_{\delta }\right))}\right]}^{-(n-1)/2}\sqrt{1+\sigma_{\delta }^{2}n_{\delta }}$	$\sqrt{1+n_{1}\sigma_{\delta }^{2}}$
	$B_{01}^{S}\approx$	$\sqrt{n}{\left(1+\frac{t^{2}}{n-2}\right)}^{-n/2}$	∞
	$B_{01}^{ZS}\approx$	$\sqrt{\frac{\pi (n-2)}{2}}{\left(1+\frac{t^{2}}{n-2}\right)}^{-(n-3)/2}$	∞
	$B_{01}^{EIA**}\approx$	$\pi^{-1}\sqrt{n_{\delta }/\left(3\hat{\eta }\right)}S^{(n-1)/2}{\left(S_{1}^{2}+S_{2}^{2}\right)}^{-(n-2)/2}\frac{\Gamma ((n-2)/2)}{\Gamma ((n-1)/2)}\times \int_{0}^{1}{\left(1-x\right)}^{-1/2}e^{-\hat{\delta }/2x}dx$	-

* Ref. [14] defines $n_{\delta }={\left(1/n_{1}+1/n_{2}\right)}^{-1}$. ** Ref. [24] defines $\hat{\eta }=\left(S_{1}^{2}+S_{2}^{2}\right)/n$ and $\hat{\delta }=2/3{\left({\bar{y}}_{1.}-{\bar{y}}_{2.}\right)}^{2}/\hat{\eta }$, and *S* is the sum of square of the two samples combined.

## Data Availability

The data presented in this study are available in [1,24].

## Abbreviations

The following abbreviations are used in this manuscript:

TESS	The effective sample size
BIC	Bayesian Information Criterion
$B_{01}^{IP}$	Bayes Factor based on the intrinsic prior for measuring $H_{0}$ vs. $H_{1}$
$B_{01}^{R}$	Bayes Factor based on the Berger’s Robust prior for measuring $H_{0}$ vs. $H_{1}$
$B_{01}^{TESS}$	Corrected BIC using the effective sample size for measuring $H_{0}$ vs. $H_{1}$
$B_{01}^{J}$	Bayes Factor based on Jeffreys’s prior for measuring $H_{0}$ vs. $H_{1}$
$B_{01}^{C}$	Bayes Factor based on the conjugate prior for measuring $H_{0}$ vs. $H_{1}$
$B_{01}^{ZS}$	Bayes Factor based on the Zellner and Siow prior for measuring $H_{0}$ vs. $H_{1}$
$B_{01}^{EIA}$	Bayes Factor expected arithmetic intrinsic prior for measuring $H_{0}$ vs. $H_{1}$

## Appendix A

### Appendix A.1. Calculation of the Effective Sample Size

We suggest using the effective sample size of [21] to define the robust Bayes factor and BIC-Tess. The effective sample size for the parameter α, $n_{o}^{e}=C^{-1}\left(X^{t}\Gamma^{-1}X\right)C^{-1}$. Let X be the design matrix:

$$X=\left[\begin{array}{cc} 1_{n\times 1} & |\left[\begin{array}{c} 1/n_{1}1_{n_{1}\times 1}\\ -1/n_{2}1_{n_{2}\times 1} \end{array}\right] \end{array}\right];$$

where $n=n_{1}+n_{2}$. Let $X_{1}$ be the second column of the design matrix $X_{1}=\left[\begin{array}{c} 1/n_{1}1_{n_{1}\times 1}\\ -1/n_{2}1_{n_{2}\times 1} \end{array}\right]$, and let $C_{1}=\max_{j}\left\{\left|\frac{1}{n_{1}\sigma }\right|,\left|-\frac{1}{n_{2}\sigma }\right|\right\}$. Further, let $\Gamma^{-1}=\sigma^{-2}I_{n\times n}$, where $I_{n\times n}$ is an identity matrix of size *n*. It follows from the definition of the effective sample size for the original α that

(A1) $$\begin{array}{cc} n_{o}^{e} & =C_{1}^{-1}\left(X_{1}^{t}\Gamma^{-1}X_{1}\right)C_{1}^{-1}\\ \; & =C_{1}^{-2}\left[\frac{\sigma^{-2}}{n_{1}},\cdots ,\frac{\sigma^{-2}}{n_{1}},-\frac{\sigma^{-2}}{n_{2}},\cdots ,-\frac{\sigma^{-2}}{n_{2}}\right]X\\ \; & =\frac{1}{C_{1}^{2}}\left[\frac{\sigma^{-2}}{n_{1}^{2}}n_{1}+\frac{\sigma^{-2}}{n_{2}^{2}}n_{2}\right]\\ \; & =\sigma^{-2}\cdot {\left(\max\left\{\left|\frac{1}{n_{1}\sigma }\right|,\left|-\frac{1}{n_{2}\sigma }\right|\right\}\right)}^{-2}\left(\frac{1}{n_{1}}+\frac{1}{n_{2}}\right)\\ \; & =\sigma^{-2}\cdot {\left(\max\left\{\left|\frac{1}{n_{1}\sigma }\right|,\left|-\frac{1}{n_{2}\sigma }\right|\right\}\right)}^{-2}n_{\delta }\\ \; & ={\left(\max\left\{\left|\frac{1}{n_{1}}\right|,\left|-\frac{1}{n_{2}}\right|\right\}\right)}^{-2}n_{\delta }. \end{array}$$

Since σ>0, $\max\left\{\left|\frac{1}{n_{1}\sigma }\right|,\left|-\frac{1}{n_{2}\sigma }\right|\right\}=\frac{1}{\sigma }\max\left\{\left|\frac{1}{n_{1}}\right|,\left|-\frac{1}{n_{2}}\right|\right\}$. Then, the final expression of the effective sample size is obtained. The definition of the unit information is $d=0.25n_{\delta }\cdot \sigma^{2}$ and for *b*,

(A2) $$b=n_{o}^{e}\cdot d=0.25\sigma^{2}\cdot {\left(\max\left\{\left|\frac{1}{n_{1}}\right|,\left|-\frac{1}{n_{2}}\right|\right\}\right)}^{-2}.$$

The factors *b* and *d* will be defined as

(A3) $$b+d=0.25\sigma^{2}\left[n_{\delta }+{\left(\max\left\{\left|\frac{1}{n_{1}}\right|,\left|-\frac{1}{n_{2}}\right|\right\}\right)}^{-2}\right]=0.25\sigma^{2}\left(b^{*}+d^{*}\right).$$

This last calculation defines the factors *b* and *d* for the Robust Bayesian Student’s *t* test, $B_{01}^{R}$.

### Appendix A.2. Simulation Experiments

In the two-sample framework, we generated 500 datasets from a random sample from a normal distribution with parameters $\mu_{1}=5,\sigma_{1}=3$; the second group was created with a combination of several parameters for the location. The mean values were $\mu_{2}\in \left\{\mu_{1},1.5\mu_{1},2\mu_{1}\right\}$ for the standard deviation $\sigma_{2}\in \left\{\sigma_{1},2\sigma_{1},3\sigma_{1}\right\}$. In the case of the Student’s *t* with ν=1 degrees of freedom, the gamma distribution was simulated using the methods of moments for the shape parameter $\alpha_{i}=\mu_{i}^{2}/\sigma_{i}^{2}$ and scale parameter $\beta_{i}=\sigma_{i}^{2}/\mu_{i}$ for i=1,2. We reported the twice natural base logarithm Bayes factors $\left(2\log\left(B_{01}\right)\right)$ for comparing the null hypothesis ($\mu_{1}=\mu_{2}$) against the alternative ($\mu_{1}\neq \mu_{2}$). This transformation allows the interpretation to be on the same scale as deviance and likelihood ratio test statistics; see Ref. [13] for a deeper discussion.

**Table A1:** Summary statistics (means ± standard deviation) for the balanced sample ($n_{1}=n_{2}=50)$ of the $2\log(B_{01})$ simulation experiments for the intrinsic priors (IP), Berger robust prior (Robust), BIC based on the effective sample size (TESS), conjugate, Jeffrey’s, Schwarz, Zellner and Siow (ZS), and the expected arithmetic intrinsic prior of [24].

			$\mu_{1}=\mu_{2}$			$\mu_{2}=1.5\mu_{1}$			$\mu_{2}=2\mu_{1}$	
		$\sigma_{1}=\sigma_{2}$	$\sigma_{2}=2\sigma_{1}$	$\sigma_{2}=3\sigma_{1}$	$\sigma_{1}=\sigma_{2}$	$\sigma_{2}=2\sigma_{1}$	$\sigma_{2}=3\sigma_{1}$	$\sigma_{1}=\sigma_{2}$	$\sigma_{2}=2\sigma_{1}$	$\sigma_{2}=3\sigma_{1}$
Normal	IP	11.3±1.59	11.44±1.37	11.42±1.24	−4.85±7.44	4.85±5.29	7.85±4.18	−41.19±11.66	−13.18±8.77	−1.53±7.19
	Robust	10.61±1.59	10.75±1.38	10.73±1.25	−5.55±7.45	4.15±5.29	7.15±4.18	−41.94±11.68	−13.9±8.78	−2.23±7.19
	TESS	9.9±1.61	10.04±1.39	10.03±1.26	−6.42±7.53	3.38±5.35	6.41±4.22	−43.18±11.8	−14.85±8.87	−3.07±7.27
	Conjugate	1.23±1.42	1.35±1.23	1.34±1.11	−13.02±6.5	−4.5±4.67	−1.84±3.7	−43.99±9.64	−20.27±7.57	−10.11±6.29
	Jeffrey’s	2.54±1.59	2.68±1.38	2.66±1.25	−13.62±7.45	−3.92±5.29	−0.91±4.18	−50.01±11.68	−21.97±8.78	−10.3±7.19
	Schwarz’s	3.47±1.61	3.61±1.39	3.59±1.26	−12.86±7.53	−3.06±5.35	−0.02±4.22	−49.62±11.8	−21.29±8.87	−9.5±7.27
	ZS	3.93±1.56	4.07±1.35	4.05±1.22	−11.9±7.3	−2.4±5.19	0.55±4.1	−47.56±11.44	−20.08±8.61	−8.65±7.05
	EIA	3.4±1.57	3.53±1.35	3.51±1.22	−12.45±7.3	−2.94±5.19	0±4.1	−47.97±11.36	−20.62±8.59	−9.2±7.04
Student-*t*(1)	IP	11.41±1.01	11.46±1.04	11.4±1.22	10.55±2.42	10.83±2.01	11.09±1.61	8.32±4.98	9.05±3.76	9.79±2.97
	Robust	10.72±1.01	10.77±1.04	10.7±1.22	9.85±2.42	10.13±2.02	10.39±1.62	7.62±4.98	8.36±3.77	9.09±2.97
	TESS	10.02±1.03	10.06±1.05	10±1.23	9.14±2.44	9.42±2.04	9.69±1.63	6.89±5.03	7.63±3.81	8.37±3
	Conjugate	1.33±0.9	1.37±0.93	1.31±1.08	0.56±2.15	0.81±1.79	1.04±1.44	−1.42±4.4	−0.77±3.34	−0.12±2.63
	Jeffrey’s	2.65±1.01	2.7±1.04	2.64±1.22	1.79±2.42	2.07±2.02	2.33±1.62	−0.44±4.98	0.29±3.77	1.03±2.97
	Schwarz’s	3.58±1.03	3.63±1.05	3.56±1.23	2.7±2.44	2.99±2.04	3.25±1.63	0.45±5.03	1.19±3.81	1.94±3
	ZS	4.04±0.99	4.09±1.02	4.03±1.19	3.19±2.37	3.47±1.98	3.72±1.58	1.01±4.88	1.73±3.69	2.45±2.91
	EIA	3.5±1	3.55±1.02	3.49±1.19	2.65±2.37	2.93±1.98	3.18±1.59	0.47±4.88	1.19±3.7	1.91±2.91
Gamma	IP	11.48±1.26	−38.13±9.19	−85.57±13.22	4.16±4.16	−5.59±5.51	−40.51±9.69	−1.33±4.72	7.9±3.41	−17.76±7.26
	Robust	10.79±1.26	−38.87±9.21	−86.4±13.25	3.46±4.16	−6.3±5.52	−41.26±9.7	−2.04±4.73	7.2±3.41	−18.48±7.27
	TESS	10.08±1.27	−40.08±9.3	−88.08±13.38	2.68±4.21	−7.18±5.57	−42.49±9.8	−2.87±4.77	6.46±3.44	−19.48±7.34
	Conjugate	1.39±1.12	−41.48±7.68	−79.22±9.99	−5.11±3.68	−13.69±4.81	−43.46±8.02	−9.96±4.15	−1.8±3.02	−24.24±6.23
	Jeffrey’s	2.72±1.26	−46.94±9.21	−94.46±13.25	−4.6±4.16	−14.37±5.52	−49.33±9.7	−10.1±4.73	−0.86±3.41	−26.55±7.27
	Schwarz	3.64±1.27	−46.52±9.3	−94.52±13.38	−3.75±4.21	−13.61±5.57	−48.93±9.8	−9.31±4.77	0.02±3.44	−25.92±7.34
	ZS	4.1±1.23	−44.55±9.02	−91.12±12.98	−3.07±4.08	−12.64±5.4	−46.89±9.51	−8.46±4.63	0.59±3.34	−24.57±7.12
	EIA	3.57±1.24	−44.99±8.97	−91.11±12.83	−3.62±4.08	−13.18±5.4	−47.32±9.44	−9.01±4.63	0.05±3.35	−25.1±7.1

**Table A2:** Frequency distribution for the balanced framework ($n_{1}=n_{2}=50)$ in the normal random variables of the $2\log(B_{01})$ based on the scale proposed by [13]. Intrinsic priors (IP), Berger robust prior (Robust), BIC based on the effective sample size (TESS), conjugate, Jeffrey’s, Schwarz, Zellner and Siow (ZS), and the expected arithmetic intrinsic prior of [24].

								$\mu_{1}=\mu_{2}$							
			$\sigma_{1}=\sigma_{2}$					$\sigma_{2}=2\sigma_{1}$					$\sigma_{2}=3\sigma_{1}$		
	Very Strong	Strong	Positive	Weak	Negative	Very Strong	Strong	Positive	Weak	Negative	Very Strong	Strong	Positive	Weak	Negative
IP	434 (86.8%)	56 (11.2%)	9 (1.8%)	1 (0.2%)	0 (0%)	439 (87.8%)	57 (11.4%)	4 (0.8%)	0 (0%)	0 (0%)	438 (87.6%)	60 (12%)	2 (0.4%)	0 (0%)	0 (0%)
Robust	400 (80%)	85 (17%)	13 (2.6%)	2 (0.4%)	0 (0%)	407 (81.4%)	85 (17%)	8 (1.6%)	0 (0%)	0 (0%)	402 (80.4%)	93 (18.6%)	5 (1%)	0 (0%)	0 (0%)
TESS	339 (67.8%)	141 (28.2%)	17 (3.4%)	3 (0.6%)	0 (0%)	344 (68.8%)	144 (28.8%)	12 (2.4%)	0 (0%)	0 (0%)	329 (65.8%)	164 (32.8%)	7 (1.4%)	0 (0%)	0 (0%)
Conjugate	0 (0%)	0 (0%)	171 (34.2%)	264 (52.8%)	65 (13%)	0 (0%)	0 (0%)	200 (40%)	241 (48.2%)	59 (11.8%)	0 (0%)	0 (0%)	182 (36.4%)	261 (52.2%)	57 (11.4%)
Jeffrey	0 (0%)	0 (0%)	392 (78.4%)	73 (14.6%)	35 (7%)	0 (0%)	0 (0%)	402 (80.4%)	68 (13.6%)	30 (6%)	0 (0%)	0 (0%)	396 (79.2%)	78 (15.6%)	26 (5.2%)
Schwarz	0 (0%)	0 (0%)	437 (87.4%)	40 (8%)	23 (4.6%)	0 (0%)	0 (0%)	444 (88.8%)	39 (7.8%)	17 (3.4%)	0 (0%)	0 (0%)	445 (89%)	45 (9%)	10 (2%)
ZS	0 (0%)	0 (0%)	452 (90.4%)	30 (6%)	18 (3.6%)	0 (0%)	0 (0%)	458 (91.6%)	31 (6.2%)	11 (2.2%)	0 (0%)	0 (0%)	459 (91.8%)	34 (6.8%)	7 (1.4%)
EIA	0 (0%)	0 (0%)	435 (87%)	42 (8.4%)	23 (4.6%)	0 (0%)	0 (0%)	444 (88.8%)	39 (7.8%)	17 (3.4%)	0 (0%)	0 (0%)	444 (88.8%)	46 (9.2%)	10 (2%)
								$\mu_{2}=1.5\mu_{1}$							
			$\sigma_{1}=\sigma_{2}$					$\sigma_{2}=2\sigma_{1}$					$\sigma_{2}=3\sigma_{1}$		
	Very Strong	Strong	Positive	Weak	Negative	Very Strong	Strong	Positive	Weak	Negative	Very Strong	Strong	Positive	Weak	Negative
IP	2 (0.4%)	19 (3.8%)	67 (13.4%)	48 (9.6%)	364 (72.8%)	69 (13.8%)	184 (36.8%)	110 (22%)	54 (10.8%)	83 (16.6%)	176 (35.2%)	195 (39%)	87 (17.4%)	21 (4.2%)	21 (4.2%)
Robust	1 (0.2%)	12 (2.4%)	60 (12%)	45 (9%)	382 (76.4%)	50 (10%)	176 (35.2%)	122 (24.4%)	52 (10.4%)	100 (20%)	137 (27.4%)	214 (42.8%)	97 (19.4%)	23 (4.6%)	29 (5.8%)
TESS	0 (0%)	11 (2.2%)	45 (9%)	45 (9%)	399 (79.8%)	24 (4.8%)	167 (33.4%)	137 (27.4%)	46 (9.2%)	126 (25.2%)	76 (15.2%)	248 (49.6%)	116 (23.2%)	26 (5.2%)	34 (6.8%)
Conjugate	0 (0%)	0 (0%)	0 (0%)	2 (0.4%)	498 (99.6%)	0 (0%)	0 (0%)	8 (1.6%)	63 (12.6%)	429 (85.8%)	0 (0%)	0 (0%)	36 (7.2%)	147 (29.4%)	317 (63.4%)
Jeffrey	0 (0%)	0 (0%)	1 (0.2%)	5 (1%)	494 (98.8%)	0 (0%)	0 (0%)	50 (10%)	77 (15.4%)	373 (74.6%)	0 (0%)	0 (0%)	132 (26.4%)	128 (25.6%)	240 (48%)
Schwarz	0 (0%)	0 (0%)	2 (0.4%)	9 (1.8%)	489 (97.8%)	0 (0%)	0 (0%)	77 (15.4%)	97 (19.4%)	326 (65.2%)	0 (0%)	0 (0%)	186 (37.2%)	117 (23.4%)	197 (39.4%)
ZS	0 (0%)	0 (0%)	3 (0.6%)	9 (1.8%)	488 (97.6%)	0 (0%)	0 (0%)	101 (20.2%)	96 (19.2%)	303 (60.6%)	0 (0%)	0 (0%)	228 (45.6%)	99 (19.8%)	173 (34.6%)
EIA	0 (0%)	0 (0%)	2 (0.4%)	9 (1.8%)	489 (97.8%)	0 (0%)	0 (0%)	74 (14.8%)	103 (20.6%)	323 (64.6%)	0 (0%)	0 (0%)	185 (37%)	119 (23.8%)	196 (39.2%)
								$\mu_{2}=2\mu_{1}$							
			$\sigma_{1}=\sigma_{2}$					$\sigma_{2}=2\sigma_{1}$					$\sigma_{2}=3\sigma_{1}$		
	Very Strong	Strong	Positive	Weak	Negative	Very Strong	Strong	Positive	Weak	Negative	Very Strong	Strong	Positive	Weak	Negative
IP	0 (0%)	0 (0%)	0 (0%)	0 (0%)	500 (100%)	0 (0%)	0 (0%)	11 (2.2%)	13 (2.6%)	476 (95.2%)	12 (2.4%)	52 (10.4%)	109 (21.8%)	53 (10.6%)	274 (54.8%)
Robust	0 (0%)	0 (0%)	0 (0%)	0 (0%)	500 (100%)	0 (0%)	0 (0%)	10 (2%)	9 (1.8%)	481 (96.2%)	5 (1%)	46 (9.2%)	102 (20.4%)	54 (10.8%)	293 (58.6%)
TESS	0 (0%)	0 (0%)	0 (0%)	0 (0%)	500 (100%)	0 (0%)	0 (0%)	5 (1%)	12 (2.4%)	483 (96.6%)	5 (1%)	38 (7.6%)	90 (18%)	53 (10.6%)	314 (62.8%)
Conjugate	0 (0%)	0 (0%)	0 (0%)	0 (0%)	500 (100%)	0 (0%)	0 (0%)	0 (0%)	0 (0%)	500 (100%)	0 (0%)	0 (0%)	1 (0.2%)	11 (2.2%)	488 (97.6%)
Jeffrey	0 (0%)	0 (0%)	0 (0%)	0 (0%)	500 (100%)	0 (0%)	0 (0%)	0 (0%)	0 (0%)	500 (100%)	0 (0%)	0 (0%)	5 (1%)	19 (3.8%)	476 (95.2%)
Schwarz	0 (0%)	0 (0%)	0 (0%)	0 (0%)	500 (100%)	0 (0%)	0 (0%)	0 (0%)	0 (0%)	500 (100%)	0 (0%)	0 (0%)	13 (2.6%)	21 (4.2%)	466 (93.2%)
ZS	0 (0%)	0 (0%)	0 (0%)	0 (0%)	500 (100%)	0 (0%)	0 (0%)	0 (0%)	0 (0%)	500 (100%)	0 (0%)	0 (0%)	18 (3.6%)	25 (5%)	457 (91.4%)
EIA	0 (0%)	0 (0%)	0 (0%)	0 (0%)	500 (100%)	0 (0%)	0 (0%)	0 (0%)	0 (0%)	500 (100%)	0 (0%)	0 (0%)	13 (2.6%)	21 (4.2%)	466 (93.2%)

**Table A3:** Frequency distribution for the balanced framework ($n_{1}=n_{2}=50)$ Student’s *t* random variables of the $2\log(B_{01})$ based on the scale proposed by [13]. Intrinsic priors (IP), Berger robust prior (Robust), BIC based on the effective sample size (TESS), conjugate, Jeffrey’s, Schwarz, Zellner and Siow (ZS), and the expected arithmetic intrinsic prior of [24].

								$\mu_{1}=\mu_{2}$							
			$\sigma_{1}=\sigma_{2}$					$\sigma_{2}=2\sigma_{1}$					$\sigma_{2}=3\sigma_{1}$		
	Very Strong	Strong	Positive	Weak	Negative	Very Strong	Strong	Positive	Weak	Negative	Very Strong	Strong	Positive	Weak	Negative
IP	456 (91.2%)	41 (8.2%)	3 (0.6%)	0 (0%)	0 (0%)	455 (91%)	44 (8.8%)	1 (0.2%)	0 (0%)	0 (0%)	448 (89.6%)	49 (9.8%)	3 (0.6%)	0 (0%)	0 (0%)
Robust	410 (82%)	87 (17.4%)	3 (0.6%)	0 (0%)	0 (0%)	416 (83.2%)	82 (16.4%)	2 (0.4%)	0 (0%)	0 (0%)	410 (82%)	83 (16.6%)	7 (1.4%)	0 (0%)	0 (0%)
TESS	310 (62%)	187 (37.4%)	3 (0.6%)	0 (0%)	0 (0%)	320 (64%)	178 (35.6%)	2 (0.4%)	0 (0%)	0 (0%)	310 (62%)	181 (36.2%)	8 (1.6%)	1 (0.2%)	0 (0%)
Conjugate	0 (0%)	0 (0%)	131 (26.2%)	326 (65.2%)	43 (8.6%)	0 (0%)	0 (0%)	156 (31.2%)	301 (60.2%)	43 (8.6%)	0 (0%)	0 (0%)	155 (31%)	297 (59.4%)	48 (9.6%)
Jeffrey	0 (0%)	0 (0%)	404 (80.8%)	85 (17%)	11 (2.2%)	0 (0%)	0 (0%)	412 (82.4%)	74 (14.8%)	14 (2.8%)	0 (0%)	0 (0%)	396 (79.2%)	86 (17.2%)	18 (3.6%)
Schwarz	0 (0%)	0 (0%)	459 (91.8%)	37 (7.4%)	4 (0.8%)	0 (0%)	0 (0%)	457 (91.4%)	39 (7.8%)	4 (0.8%)	0 (0%)	0 (0%)	454 (90.8%)	36 (7.2%)	10 (2%)
ZS	0 (0%)	0 (0%)	479 (95.8%)	18 (3.6%)	3 (0.6%)	0 (0%)	0 (0%)	474 (94.8%)	24 (4.8%)	2 (0.4%)	0 (0%)	0 (0%)	474 (94.8%)	17 (3.4%)	9 (1.8%)
EIA	0 (0%)	0 (0%)	457 (91.4%)	39 (7.8%)	4 (0.8%)	0 (0%)	0 (0%)	457 (91.4%)	39 (7.8%)	4 (0.8%)	0 (0%)	0 (0%)	454 (90.8%)	36 (7.2%)	10 (2%)
								$\mu_{2}=1.5\mu_{1}$							
			$\sigma_{1}=\sigma_{2}$					$\sigma_{2}=2\sigma_{1}$					$\sigma_{2}=3\sigma_{1}$		
	Very Strong	Strong	Positive	Weak	Negative	Very Strong	Strong	Positive	Weak	Negative	Very Strong	Strong	Positive	Weak	Negative
IP	378 (75.6%)	92 (18.4%)	22 (4.4%)	5 (1%)	3 (0.6%)	395 (79%)	88 (17.6%)	14 (2.8%)	1 (0.2%)	2 (0.4%)	421 (84.2%)	69 (13.8%)	10 (2%)	0 (0%)	0 (0%)
Robust	327 (65.4%)	132 (26.4%)	31 (6.2%)	5 (1%)	5 (1%)	347 (69.4%)	129 (25.8%)	19 (3.8%)	3 (0.6%)	2 (0.4%)	375 (75%)	111 (22.2%)	14 (2.8%)	0 (0%)	0 (0%)
TESS	259 (51.8%)	187 (37.4%)	39 (7.8%)	9 (1.8%)	6 (1.2%)	271 (54.2%)	199 (39.8%)	23 (4.6%)	4 (0.8%)	3 (0.6%)	284 (56.8%)	191 (38.2%)	24 (4.8%)	1 (0.2%)	0 (0%)
Conjugate	0 (0%)	0 (0%)	125 (25%)	259 (51.8%)	116 (23.2%)	0 (0%)	0 (0%)	109 (21.8%)	289 (57.8%)	102 (20.4%)	0 (0%)	0 (0%)	139 (27.8%)	283 (56.6%)	78 (15.6%)
Jeffrey	0 (0%)	0 (0%)	321 (64.2%)	102 (20.4%)	77 (15.4%)	0 (0%)	0 (0%)	342 (68.4%)	100 (20%)	58 (11.6%)	0 (0%)	0 (0%)	366 (73.2%)	90 (18%)	44 (8.8%)
Schwarz	0 (0%)	0 (0%)	387 (77.4%)	53 (10.6%)	60 (12%)	0 (0%)	0 (0%)	403 (80.6%)	60 (12%)	37 (7.4%)	0 (0%)	0 (0%)	424 (84.8%)	47 (9.4%)	29 (5.8%)
ZS	0 (0%)	0 (0%)	403 (80.6%)	46 (9.2%)	51 (10.2%)	0 (0%)	0 (0%)	426 (85.2%)	44 (8.8%)	30 (6%)	0 (0%)	0 (0%)	443 (88.6%)	32 (6.4%)	25 (5%)
EIA	0 (0%)	0 (0%)	387 (77.4%)	53 (10.6%)	60 (12%)	0 (0%)	0 (0%)	401 (80.2%)	63 (12.6%)	36 (7.2%)	0 (0%)	0 (0%)	423 (84.6%)	49 (9.8%)	28 (5.6%)
								$\mu_{2}=2\mu_{1}$							
			$\sigma_{1}=\sigma_{2}$					$\sigma_{2}=2\sigma_{1}$					$\sigma_{2}=3\sigma_{1}$		
	Very Strong	Strong	Positive	Weak	Negative	Very Strong	Strong	Positive	Weak	Negative	Very Strong	Strong	Positive	Weak	Negative
IP	264 (52.8%)	125 (25%)	60 (12%)	12 (2.4%)	39 (7.8%)	263 (52.6%)	144 (28.8%)	64 (12.8%)	11 (2.2%)	18 (3.6%)	324 (64.8%)	114 (22.8%)	48 (9.6%)	7 (1.4%)	7 (1.4%)
Robust	225 (45%)	150 (30%)	65 (13%)	18 (3.6%)	42 (8.4%)	231 (46.2%)	166 (33.2%)	72 (14.4%)	9 (1.8%)	22 (4.4%)	270 (54%)	157 (31.4%)	55 (11%)	8 (1.6%)	10 (2%)
TESS	162 (32.4%)	190 (38%)	79 (15.8%)	22 (4.4%)	47 (9.4%)	183 (36.6%)	196 (39.2%)	81 (16.2%)	16 (3.2%)	24 (4.8%)	183 (36.6%)	229 (45.8%)	64 (12.8%)	10 (2%)	14 (2.8%)
Conjugate	0 (0%)	0 (0%)	73 (14.6%)	194 (38.8%)	233 (46.6%)	0 (0%)	0 (0%)	73 (14.6%)	193 (38.6%)	234 (46.8%)	0 (0%)	0 (0%)	82 (16.4%)	245 (49%)	173 (34.6%)
Jeffrey	0 (0%)	0 (0%)	224 (44.8%)	87 (17.4%)	189 (37.8%)	0 (0%)	0 (0%)	229 (45.8%)	102 (20.4%)	169 (33.8%)	0 (0%)	0 (0%)	261 (52.2%)	110 (22%)	129 (25.8%)
Schwarz	0 (0%)	0 (0%)	268 (53.6%)	77 (15.4%)	155 (31%)	0 (0%)	0 (0%)	269 (53.8%)	98 (19.6%)	133 (26.6%)	0 (0%)	0 (0%)	330 (66%)	75 (15%)	95 (19%)
ZS	0 (0%)	0 (0%)	287 (57.4%)	72 (14.4%)	141 (28.2%)	0 (0%)	0 (0%)	298 (59.6%)	82 (16.4%)	120 (24%)	0 (0%)	0 (0%)	349 (69.8%)	64 (12.8%)	87 (17.4%)
EIA	0 (0%)	0 (0%)	268 (53.6%)	78 (15.6%)	154 (30.8%)	0 (0%)	0 (0%)	268 (53.6%)	100 (20%)	132 (26.4%)	0 (0%)	0 (0%)	330 (66%)	77 (15.4%)	93 (18.6%)

**Table A4:** Frequency distribution for the balanced framework ($n_{1}=n_{2}=50)$ from the gamma random variables of the $2\log(B_{01})$ based on the scale proposed by [13]. Intrinsic priors (IP), Berger robust prior (Robust), BIC based on the effective sample size (TESS), conjugate, Jeffrey’s, Schwarz, Zellner and Siow (ZS), and the expected arithmetic intrinsic prior of [24].

								$\mu_{1}=\mu_{2}$							
			$\sigma_{1}=\sigma_{2}$					$\sigma_{2}=2\sigma_{1}$					$\sigma_{2}=3\sigma_{1}$		
	Very Strong	Strong	Positive	Weak	Negative	Very Strong	Strong	Positive	Weak	Negative	Very Strong	Strong	Positive	Weak	Negative
IP	449 (89.8%)	49 (9.8%)	2 (0.4%)	0 (0%)	0 (0%)	0 (0%)	0 (0%)	0 (0%)	0 (0%)	500 (100%)	0 (0%)	0 (0%)	0 (0%)	0 (0%)	500 (100%)
Robust	417 (83.4%)	78 (15.6%)	5 (1%)	0 (0%)	0 (0%)	0 (0%)	0 (0%)	0 (0%)	0 (0%)	500 (100%)	0 (0%)	0 (0%)	0 (0%)	0 (0%)	500 (100%)
TESS	348 (69.6%)	139 (27.8%)	13 (2.6%)	0 (0%)	0 (0%)	0 (0%)	0 (0%)	0 (0%)	0 (0%)	500 (100%)	0 (0%)	0 (0%)	0 (0%)	0 (0%)	500 (100%)
Conjugate	0 (0%)	0 (0%)	183 (36.6%)	268 (53.6%)	49 (9.8%)	0 (0%)	0 (0%)	0 (0%)	0 (0%)	500 (100%)	0 (0%)	0 (0%)	0 (0%)	0 (0%)	500 (100%)
Jeffrey	0 (0%)	0 (0%)	412 (82.4%)	62 (12.4%)	26 (5.2%)	0 (0%)	0 (0%)	0 (0%)	0 (0%)	500 (100%)	0 (0%)	0 (0%)	0 (0%)	0 (0%)	500 (100%)
Schwarz	0 (0%)	0 (0%)	452 (90.4%)	31 (6.2%)	17 (3.4%)	0 (0%)	0 (0%)	0 (0%)	0 (0%)	500 (100%)	0 (0%)	0 (0%)	0 (0%)	0 (0%)	500 (100%)
ZS	0 (0%)	0 (0%)	465 (93%)	25 (5%)	10 (2%)	0 (0%)	0 (0%)	0 (0%)	0 (0%)	500 (100%)	0 (0%)	0 (0%)	0 (0%)	0 (0%)	500 (100%)
EIA	0 (0%)	0 (0%)	451 (90.2%)	32 (6.4%)	17 (3.4%)	0 (0%)	0 (0%)	0 (0%)	0 (0%)	500 (100%)	0 (0%)	0 (0%)	0 (0%)	0 (0%)	500 (100%)
								$\mu_{2}=1.5\mu_{1}$							
			$\sigma_{1}=\sigma_{2}$					$\sigma_{2}=2\sigma_{1}$					$\sigma_{2}=3\sigma_{1}$		
	Very Strong	Strong	Positive	Weak	Negative	Very Strong	Strong	Positive	Weak	Negative	Very Strong	Strong	Positive	Weak	Negative
IP	30 (6%)	141 (28.2%)	186 (37.2%)	63 (12.6%)	80 (16%)	0 (0%)	3 (0.6%)	33 (6.6%)	35 (7%)	429 (85.8%)	0 (0%)	0 (0%)	0 (0%)	0 (0%)	500 (100%)
Robust	21 (4.2%)	119 (23.8%)	191 (38.2%)	68 (13.6%)	101 (20.2%)	0 (0%)	0 (0%)	22 (4.4%)	39 (7.8%)	439 (87.8%)	0 (0%)	0 (0%)	0 (0%)	0 (0%)	500 (100%)
TESS	8 (1.6%)	109 (21.8%)	186 (37.2%)	72 (14.4%)	125 (25%)	0 (0%)	0 (0%)	16 (3.2%)	33 (6.6%)	451 (90.2%)	0 (0%)	0 (0%)	0 (0%)	0 (0%)	500 (100%)
Conjugate	0 (0%)	0 (0%)	2 (0.4%)	32 (6.4%)	466 (93.2%)	0 (0%)	0 (0%)	0 (0%)	0 (0%)	500 (100%)	0 (0%)	0 (0%)	0 (0%)	0 (0%)	500 (100%)
Jeffrey	0 (0%)	0 (0%)	19 (3.8%)	49 (9.8%)	432 (86.4%)	0 (0%)	0 (0%)	0 (0%)	0 (0%)	500 (100%)	0 (0%)	0 (0%)	0 (0%)	0 (0%)	500 (100%)
Schwarz	0 (0%)	0 (0%)	36 (7.2%)	63 (12.6%)	401 (80.2%)	0 (0%)	0 (0%)	0 (0%)	0 (0%)	500 (100%)	0 (0%)	0 (0%)	0 (0%)	0 (0%)	500 (100%)
ZS	0 (0%)	0 (0%)	50 (10%)	73 (14.6%)	377 (75.4%)	0 (0%)	0 (0%)	0 (0%)	0 (0%)	500 (100%)	0 (0%)	0 (0%)	0 (0%)	0 (0%)	500 (100%)
EIA	0 (0%)	0 (0%)	35 (7%)	64 (12.8%)	401 (80.2%)	0 (0%)	0 (0%)	0 (0%)	0 (0%)	500 (100%)	0 (0%)	0 (0%)	0 (0%)	0 (0%)	500 (100%)
								$\mu_{2}=2\mu_{1}$							
			$\sigma_{1}=\sigma_{2}$					$\sigma_{2}=2\sigma_{1}$					$\sigma_{2}=3\sigma_{1}$		
	Very Strong	Strong	Positive	Weak	Negative	Very Strong	Strong	Positive	Weak	Negative	Very Strong	Strong	Positive	Weak	Negative
IP	0 (0%)	19 (3.8%)	103 (20.6%)	86 (17.2%)	292 (58.4%)	148 (29.6%)	218 (43.6%)	103 (20.6%)	17 (3.4%)	14 (2.8%)	0 (0%)	0 (0%)	0 (0%)	0 (0%)	500 (100%)
Robust	0 (0%)	13 (2.6%)	87 (17.4%)	73 (14.6%)	327 (65.4%)	111 (22.2%)	229 (45.8%)	122 (24.4%)	16 (3.2%)	22 (4.4%)	0 (0%)	0 (0%)	0 (0%)	0 (0%)	500 (100%)
TESS	0 (0%)	6 (1.2%)	71 (14.2%)	66 (13.2%)	357 (71.4%)	73 (14.6%)	239 (47.8%)	140 (28%)	21 (4.2%)	27 (5.4%)	0 (0%)	0 (0%)	0 (0%)	0 (0%)	500 (100%)
Conjugate	0 (0%)	0 (0%)	0 (0%)	0 (0%)	500 (100%)	0 (0%)	0 (0%)	28 (5.6%)	124 (24.8%)	348 (69.6%)	0 (0%)	0 (0%)	0 (0%)	0 (0%)	500 (100%)
Jeffrey	0 (0%)	0 (0%)	0 (0%)	2 (0.4%)	498 (99.6%)	0 (0%)	0 (0%)	107 (21.4%)	130 (26%)	263 (52.6%)	0 (0%)	0 (0%)	0 (0%)	0 (0%)	500 (100%)
Schwarz	0 (0%)	0 (0%)	0 (0%)	5 (1%)	495 (99%)	0 (0%)	0 (0%)	157 (31.4%)	139 (27.8%)	204 (40.8%)	0 (0%)	0 (0%)	0 (0%)	0 (0%)	500 (100%)
ZS	0 (0%)	0 (0%)	0 (0%)	7 (1.4%)	493 (98.6%)	0 (0%)	0 (0%)	200 (40%)	115 (23%)	185 (37%)	0 (0%)	0 (0%)	0 (0%)	0 (0%)	500 (100%)
EIA	0 (0%)	0 (0%)	0 (0%)	5 (1%)	495 (99%)	0 (0%)	0 (0%)	154 (30.8%)	143 (28.6%)	203 (40.6%)	0 (0%)	0 (0%)	0 (0%)	0 (0%)	500 (100%)
